# Metatranscriptomics reveals a differential temperature effect on the structural and functional organization of the anaerobic food web in rice field soil

**DOI:** 10.1186/s40168-018-0546-9

**Published:** 2018-09-19

**Authors:** Jingjing Peng, Carl-Eric Wegner, Qicheng Bei, Pengfei Liu, Werner Liesack

**Affiliations:** 1 Research Group Methanotrophic Bacteria and Environmental Genomics/Transcriptomics, Max Planck Institute for Terrestrial Microbiology, Karl-von-Frisch-Str. 10, 35043 Marburg, Germany; 2 Institute of Ecology, Aquatic Geomicrobiology, Friedrich Schiller University Jena, Dornburger Str. 159, 07749 Jena, Germany

**Keywords:** rRNA, mRNA, Biopolymer degradation, CAZymes, Syntrophy, Formyltetrahydrofolate synthetase, Methanogenesis, *Methanosarcina*, *Methanocella*

## Abstract

**Background:**

The expected increase in global surface temperature due to climate change may have a tremendous effect on the structure and function of the anaerobic food web in flooded rice field soil. Here, we used the metatranscriptomic analysis of total RNA to gain a system-level understanding of this temperature effect on the methanogenic food web.

**Results:**

Mesophilic (30 °C) and thermophilic (45 °C) food web communities had a modular structure. Family-specific rRNA dynamics indicated that each network module represents a particular function within the food webs. Temperature had a differential effect on all the functional activities, including polymer hydrolysis, syntrophic oxidation of key intermediates, and methanogenesis. This was further evidenced by the temporal expression patterns of total bacterial and archaeal mRNA and of transcripts encoding carbohydrate-active enzymes (CAZymes). At 30 °C, various bacterial phyla contributed to polymer hydrolysis, with *Firmicutes* decreasing and non-*Firmicutes* (e.g., *Bacteroidetes*, *Ignavibacteriae*) increasing with incubation time. At 45 °C, CAZyme expression was solely dominated by the *Firmicutes* but, depending on polymer and incubation time, varied on family level. The structural and functional community dynamics corresponded well to process measurements (acetate, propionate, methane). At both temperatures, a major change in food web functionality was linked to the transition from the early to late stage. The mesophilic food web was characterized by gradual polymer breakdown that governed acetoclastic methanogenesis (*Methanosarcinaceae*) and, with polymer hydrolysis becoming the rate-limiting step, syntrophic propionate oxidation (*Christensenellaceae*, *Peptococcaceae*). The thermophilic food web had two activity stages characterized first by polymer hydrolysis and followed by syntrophic oxidation of acetate (*Thermoanaerobacteraceae*, *Heliobacteriaceae*, clade OPB54). Hydrogenotrophic *Methanocellaceae* were the syntrophic methanogen partner, but their population structure differed between the temperatures. Thermophilic temperature promoted proliferation of a new *Methanocella* ecotype.

**Conclusions:**

Temperature had a differential effect on the structural and functional continuum in which the methanogenic food web operates. This temperature-induced change in food web functionality may not only be a near-future scenario for rice paddies but also for natural wetlands in the tropics and subtropics.

**Electronic supplementary material:**

The online version of this article (10.1186/s40168-018-0546-9) contains supplementary material, which is available to authorized users.

## Background

Methane (CH_4_) is a potent greenhouse gas. On a molecule basis, it is 72 times more effective than carbon dioxide in trapping heat radiation over a 20-year period [1]. Wetland rice farming is a major source of atmospheric methane, accounting for 10% of the global CH_4_ budget (e.g., [2]). The methanogenic degradation pathway of organic matter in submerged rice paddies and anoxic wetlands follows common principles and involves a microbial food web composed of different functional guilds of the domains *Bacteria* and *Archaea* [3]. These microbial guilds participate in a cascade of anaerobic degradation steps that involve polymer hydrolysis, fermentation, syntrophic conversion of fatty acids, homoacetogenesis, and methanogenesis [4]. Initially, methanogenic degradation may be accomplished by anaerobic respiration (e.g., NO_3_^−^, Mn(IV), Fe(III) and SO_4_^2−^) but the alternative terminal electron acceptors are completely used up during the first few days under anoxic conditions [4–6].

The methanogenic community in rice field soil produces CH_4_ over a wide range up to 55 °C, with temperature being the key factor controlling CH_4_ production [7, 8]. Temperature was found not only to affect the rates of methane production but also the composition and function of methanogenic communities in rice field soils in Italy, China, Japan, Philippines, and Thailand [9–14]. Changes in both the pathways of CH_4_ production and the composition of the methanogenic community are only gradual at a mesophilic temperature range of up to 37 °C. Within this temperature range, methane production is characterized by acetoclastic and hydrogenotrophic methanogenesis [10]; however, the communities were shown to markedly change their methanogenic activities at temperatures higher than 40–42 °C [7, 14–16]. At these thermophilic temperatures, methane production will be dominated solely by hydrogenotrophic methanogenesis [7]. The same temperature-induced change was shown to also occur in Chinese natural wetlands [15]. Although the in situ temperature in paddy soil is generally lower than or around 30 °C [17], present-day temperatures above 40 °C are reached in the rice fields of southeastern China [18]. Moreover, the expected increase in global surface temperature by 2–4 °C by 2050 [1] makes temperature effects on structure and function of soil microbial communities an important research issue [19].

No study has yet been performed to gain a system-level understanding of the temperature effect on the methanogenic food web in rice field soil. In particular, the knowledge of microbial populations involved in polymer hydrolysis is limited. Previous studies were mostly based on total DNA and coupled the analysis of PCR amplicons with T-RFLP analysis or sequencing. Thus, each of these fingerprinting studies could focus only on a particular aspect of the temperature effect on community composition and structure [10, 14, 15, 20]. Along with process measurements, these studies showed that acetate and propionate are the key intermediates of the polymer breakdown in rice field soil. While syntrophic oxidation of propionate is actively occurring at low temperatures [21–23], syntrophic oxidation of acetate is thermodynamically feasible only at thermophilic temperatures [20]. Members of *Thermoacetogenium* and other *Thermoanaerobacteraceae*, but also *Symbiobacterium*, were shown to contribute to syntrophic acetate oxidation in rice field soil at 50 °C, with *Methanocellaceae* as the hydrogenotrophic partner [20, 24].

Here, we applied rRNA- and mRNA-based metatranscriptomics to assess the effect of temperature on the structural and functional organization of the anaerobic food web in rice field soil. The objectives were to (i) identify the microbial populations and functional genes involved in key metabolic processes and (ii) elucidate structure-function relationships that determine the anaerobic decomposition of plant polymers at mesophilic (30 °C) and thermophilic (45 °C) temperatures. In particular, we aimed to identify structural and functional links between polymer breakdown, syntrophy, and methanogenesis. Field conditions are known to be very complex, given that CH_4_ is produced from three different sources of organic matter: rice straw, root exudates, and soil organic matter. Their relative contributions change with time of the season [25–28]. Rice straw is the major source in the early season, while root exudates become important with the development of rice plants [25–28]. Hence, the effect of temperature on polymer breakdown and methanogenic pathways cannot be determined unambiguously under heterogenous field conditions. Therefore, we studied the impact of temperature on food web functionality under controlled laboratory conditions, with rice straw as the major carbon source. Anoxic paddy soil slurries were incubated for 30 days. Triplicate sampling for process measurements and random cDNA library construction (total of 30 libraries) were made after 5, 11, 16, 23, and 30 days of incubation.

## Results

### Co-occurrence networks

**Fig. 1:**
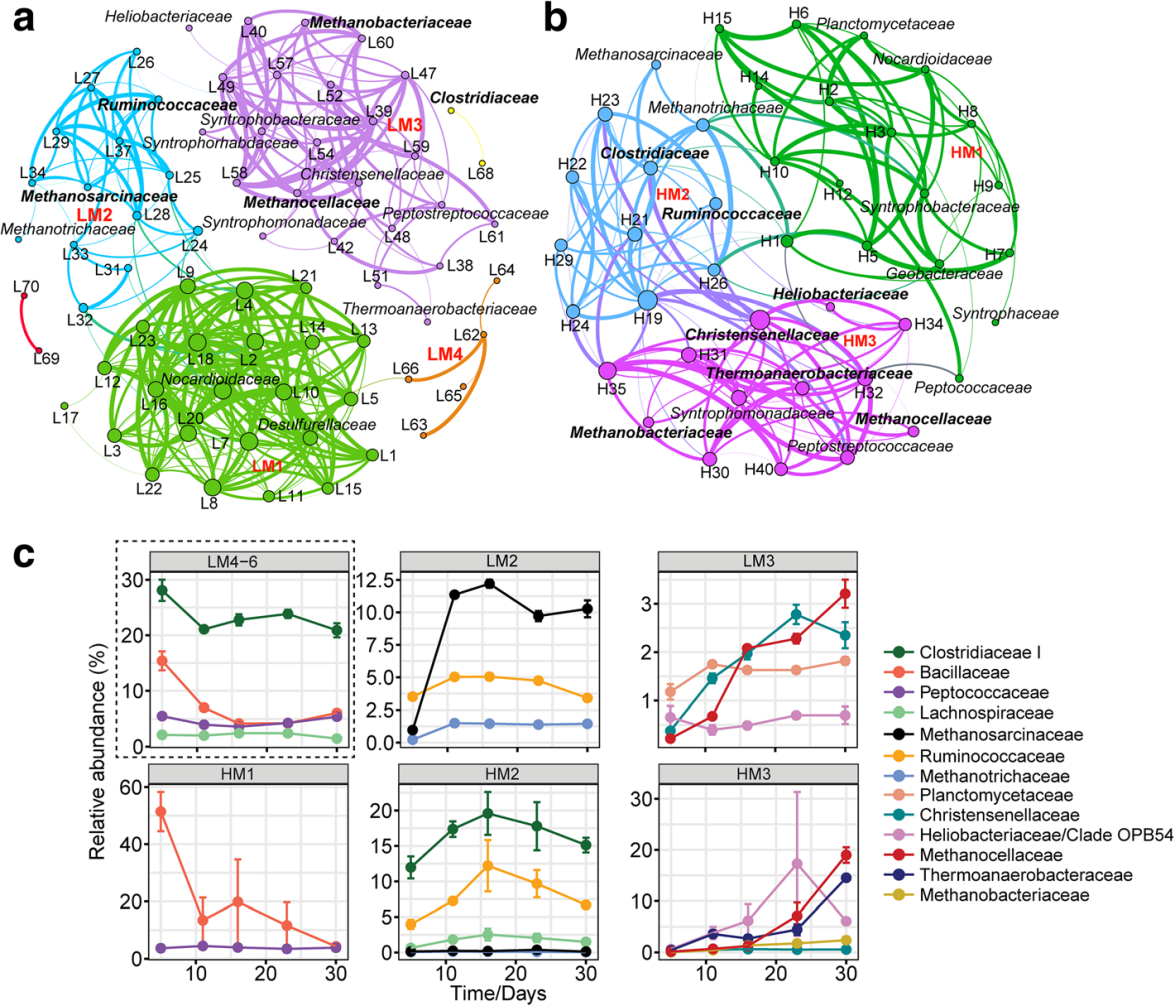
Co-occurrence network analyses for 30 °C (**a**) and 45 °C (**b**). Nodes were colored by modularity class with labeled family names. Each connection (edge) between two nodes stands for a strong (Spearman’s 0.6 ≤ *ρ* ≤ 0.9) and significant (FDR corrected *P* value < 0.01) correlation. The size of each node is proportional to the number of connections (its degree). Designation of the major modules (LM, low-temperature module; HM, high-temperature module) is shown in red bold color. (**c**) Temporal dynamics of dominant families (> 1%) in different modules at 30 °C and 45 °C

Two correlation networks were constructed based on the 16S rRNA reads assigned to each family-level group (Fig. 1a, b)*.* The correlation networks consisted of 70 nodes (30 °C) and 42 nodes (45 °C) of significant (FDR corrected, *P* ≤ 0.01) and robust (0.6 ≤ *ρ* ≤ 0.9) positive correlations (Additional file 1: Tables S1 and S2). Modularity indices were 0.57 (30 °C) and 0.47 (45 °C), indicating that both correlation networks had a modular structure [29]. Three highly complex modules were identified for both temperatures 30 °C (LM1, 2, 3; Fig. 1a) and 45 °C (HM1, 2, 3; Fig. 1b). Their taxonomic composition was linked to abundance information (Fig. 1c).

LM1 and HM1 were composed of taxa belonging to a variety of bacterial classes. Both modules contained members of the *Bacilli*, *Clostridia*, *Actinobacteria*, *Alphaproteobacteria*, and *Deltaproteobacteria* (Additional file 1: Tables S1 and S2). The relative abundance of these mostly minor populations decreased with incubation time. Particular population dynamics were observed for *Clostridium* cluster XVIII (30 °C) and *Bacillaceae* (45 °C) (Additional file 1: Figures S1 and S2). The abundance of *Clostridium* cluster XVIII was stable for the first 11 days at 30 °C but rapidly decreased thereafter. *Bacillaceae* rapidly declined in abundance during the first 11 days of incubation at 45 °C but showed a second peak abundance on day 16.

LM2 and HM2 had in common that the peak abundance of most members was around day 16 and both modules contained acetoclastic methanogens (*Methanosarcinaceae* and *Methanotrichaceae* [formerly *Methanosaetaceae*]). However, LM2 and HM2 differed in module structure. LM2 was characterized by the dominant presence of the *Methanosarcinaceae* and, to a lesser extent, *Ruminococcaceae*. Instead, HM2 was characterized by the dominant presence of *Clostridium* cluster I, *Ruminococcaceae*, *Halobacteroidaceae*, *Lachnospiraceae*, and *Limnochordaceae*. Unlike all other abundant family-level groups co-occurring in HM2, *Halobacteroidaceae* showed a distinct peak abundance on day 9. Methanogens (*Methanosarcinaceae*, *Methanotrichaceae*) were only of minor abundance (< 0.5%) (Fig. 1c and Additional file 1: Figures S1 and S2).

LM3 and HM3 had family-level groups in common that are known to be fermenters, syntrophs, or hydrogenotrophic methanogens. Among these were *Christensenellaceae*, *Syntrophomonadaceae*, *Heliobacteriaceae*, *Thermoanaerobacteraceae*, *Methanobacteriaceae*, and *Methanocellaceae*. Despite the similarities in taxonomic composition, LM3 and HM3 significantly differed in module structure (as previously observed for LM2/HM2). In addition to the *Methanocellaceae*, the most abundant family-level groups in LM3 were the *Christensenellaceae* and, to a lesser extent, *Planctomycetaceae* and *Solibacteraceae* subgroup 3 (*Acidobacteria*) (Additional file 1: Tables S1 and S2). The abundance of *Methanocellaceae* increased to 3.2% (LM3) and 19% (HM3) till day 30 (Fig. 1c and Additional file 1: Figures S1 and S2). *Heliobacteriaceae* and *Thermoanaerobacteraceae* were dominant members of HM3 but differed in their temporal dynamics. *Heliobacteriaceae* had the peak abundance around day 23 (17.3%) and decreased thereafter (7.6% [day 30]). *Thermoanaerobacteraceae* showed a highly significant co-occurrence correlation (*P* < 0.0009) with the *Methanocellaceae* and a peak abundance (14.5%) on day 30. Other taxa that showed peak abundances on day 30 were *Syntrophomonadaceae* (1.2%) and *Methanobacteriaceae* (2.4%) (Fig. 1c).

The three highly complex LM modules were distinctly separated, but they collectively represented only a major subset of total mesophilic food web community. A few abundant family-level groups did not cluster with LM1, LM2, or LM3. These were *Clostridium* cluster I, *Bacillaceae*, *Peptococcaceae*, and *Lachnospiraceae*. These families were grouped into the low-complexity modules LM4 to LM6. Collectively, their total rRNA abundance ranged from 34.1% (day 11) to 33.9% (day 30) (Fig. 1c and Additional file 1: Tables S1 and S2). At thermophilic temperature, all the family-level groups were affiliated with one of the three HM modules, including *Bacillaceae* and *Peptococcaceae* (HM1) as well as *Clostridium* cluster I and *Lachnospiraceae* (HM2). Unlike LM2/LM3, HM2 and HM3 were connected by a strong co-occurrence link. This link was related to the central network position of the *Christensenellaceae* between HM2 and HM3.

### Functional gene expression

NMDS plots on rRNA and mRNA level confirmed that temperature (*P* < 0.001) and incubation time (*P* < 0.001) had significant effects on community composition (Additional file 1: Figure S3). The phylum-level composition between total rRNA and mRNA agreed well at both temperatures 30 °C and 45 °C; however, some discrepancies were evident on family level. In particular, methanogen mRNA was significantly overrepresented relative to methanogen rRNA (Additional file 1: Figure S4).

**Fig. 2:**
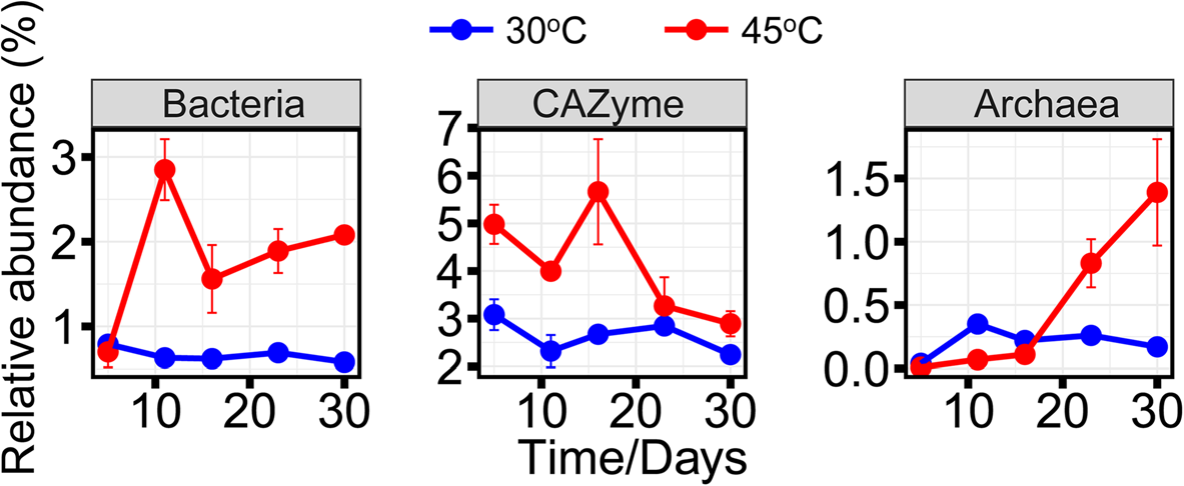
The percentage proportion of bacterial and archaeal mRNA in total RNA reads and CAZyme transcripts in total mRNA reads

The temporal dynamics of community-wide mRNA expression significantly differed between 30 and 45 °C (Fig. 2). At mesophilic temperature, bacterial and archaeal mRNA showed a low, but nearly constant, expression level throughout incubation. Collectively, their proportion of total RNA ranged between 0.04 and 0.79%. At thermophilic temperature, bacterial and archaeal mRNA expression changed with incubation time. Bacterial mRNA expression peaked on day 11, decreased towards day 16, and increased again gradually until day 30. Archaeal mRNA abundance slightly increased during the first 16 days, but its expression level greatly increased during the later stage. The proportion of bacterial and archaeal mRNA in total RNA ranged from 0.70% to 2.85% (bacteria) and 0.01% to 1.39% (archaea). Thus, community-wide mRNA expression was significantly increased relative to 30 °C.

The temporal expression dynamics of carbohydrate-active enzymes (CAZymes) also significantly differed between 30 and 45 °C. At mesophilic temperature, the proportion of CAZyme transcripts in total mRNA stably ranged between 2.24 and 3.08% throughout incubation time. At thermophilic temperature, their proportion in total mRNA was as high as 2.89% to 5.66%, with peak abundance on day 16. Thereafter, the CAZyme transcript level steadily decreased till day 30.

### Plant polymer breakdown

**Fig. 3:**
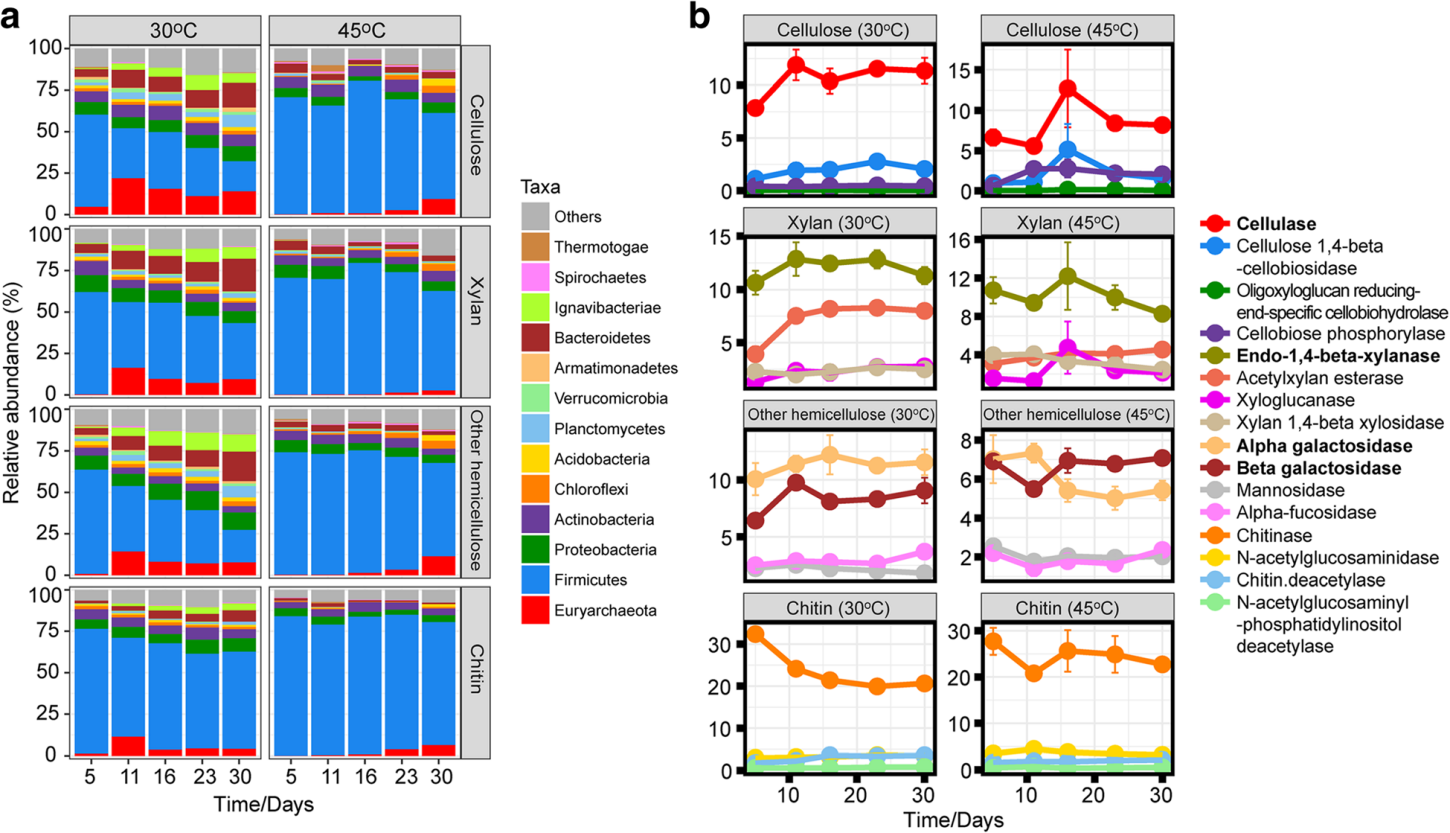
Expression dynamics of GH/CBM transcripts affiliated with particular phyla (> 0.5%) (**a**) and temporal dynamics of gene expression related to the breakdown of cellulose, xylan, other hemicelluloses, and chitin (**b**). The corresponding mRNA transcript numbers are shown in Tables S3 and S4

Totally, 92,508 mRNA reads were identified to encode CAZymes. Of these, 75,438 reads were linked to enzymes involved in the degradation of cellulose, xylan, other hemicelluloses, and chitin (Additional file 1: Table S3). These enzymes were primarily categorized into glycosyl hydrolases (GHs) and carbohydrate-binding modules (CBMs) (Fig. 3).

Both temperature and incubation time had a significant impact on the taxonomic composition of the GH/CBM transcript pool. At mesophilic temperature, various microbial phyla contributed to the expression of GHs and CBMs. The majority of GH/CBM transcripts was affiliated with the *Firmicutes*; however, their relative abundance steadily decreased with incubation time, while that of the *Bacteroidetes* and *Ignavibacteriae* increased. At thermophilic temperature, the phylum-level diversity of GH/CBM transcripts was reduced and nearly exclusively related to the *Firmicutes* (Fig. 3a). On family level, the assignment rate of GH/CBM transcripts depended on the polymer, sampling time point, and temperature, and varied between 57 and 89% (Additional file 1: Table S3). The bacterial composition of the GH/CBM transcripts greatly differed for each of the polymers between 30 and 45 °C, but all the abundant family-level groups (cutoff ≥ 0.5% of total GH/CBM transcripts) belonged to the *Firmicutes* (Additional file 1: Figure S5).

Key enzymes involved in rice straw degradation are cellulase, endo-1,4-beta-xylanase, and alpha- and beta-galactosidases (Fig. 3b and Additional file 1: Table S4). Transcripts affiliated with four family-level groups were detected to contribute to the production of all the key enzymes, with transcripts affiliated with *Methanosarcinaceae* being predominant at 30 °C, and *Clostridiaceae*, *Ruminococcaceae*, and *Paenibacillaceae* being most abundant in the GH/CBM transcript pool at 45 °C (Additional file 1: Figure S5). The potential expression of GH transcripts by *Methanosarcinaceae* was supported by querying 88 methanogen genome sequences against dbCAN. Of a total of 214,249 coding genes, 2691 were functionally annotated to encode GHs, with some of them showing 100% sequence identity to GH families linked to the breakdown of complex polysaccharides (Additional file 1: Figure S5 and Additional file 2).

### Acetate, propionate, hydrogen, and methane

**Fig. 4:**
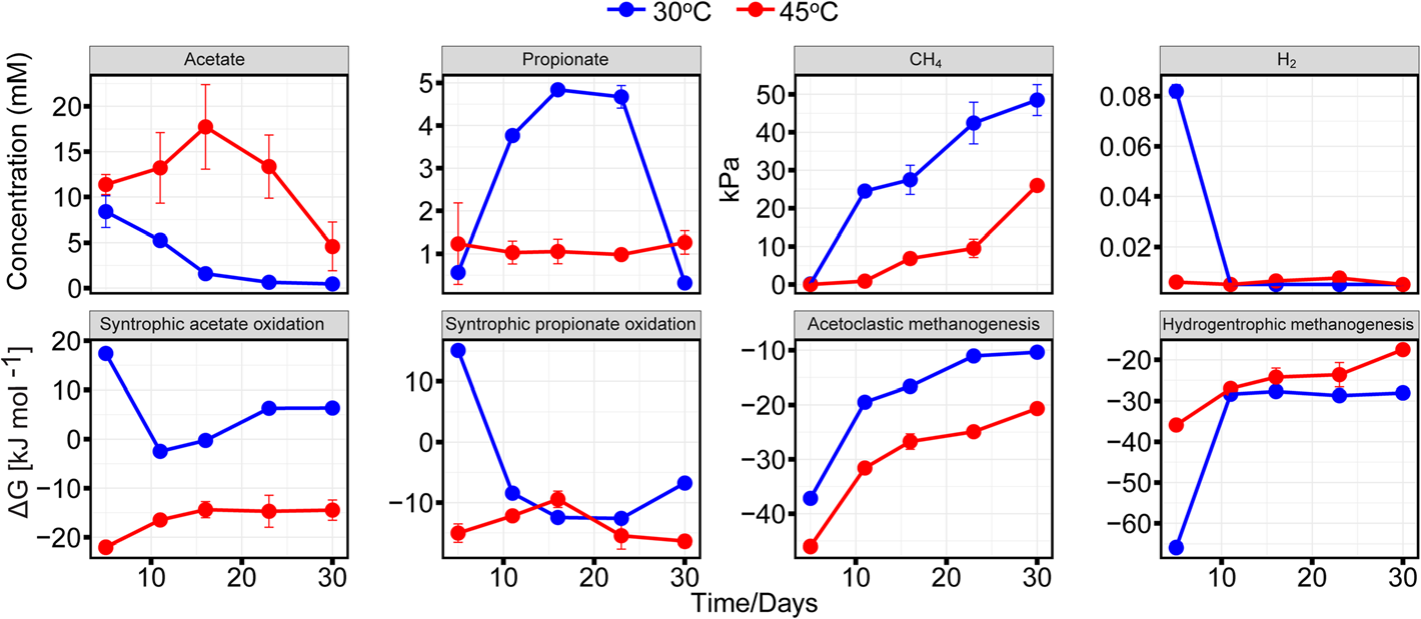
Dynamics of acetate, propionate, H_2_, and CH_4_, and Gibbs free energy (Δ*G*) of different metabolic pathways during the 30-day incubation at 30 °C and 45 °C (means ± SE, *n* = 3)

At mesophilic temperature, acetate reached its peak concentration (8.4 mM) at day 5 or shortly thereafter and then steadily decreased towards days 16 (1.6 mM) and 30 (0.5 mM) (Fig. 4). Propionate strongly accumulated from day 5 (0.6 mM) onwards, with a peak concentration between days 16 (4.8 mM) and 23 (4.7 mM). Thereafter, its concentration rapidly decreased within 7 days to 0.3 mM on day 30. At thermophilic temperature, acetate accumulated to significantly higher concentrations than at 30 °C. It rapidly accumulated to 11.4 mM on day 5 and reached its peak concentration (17.7 mM) on day 16. Thereafter, the acetate concentration gradually decreased to 4.6 mM on day 30. The propionate concentration reached a steady state between production and consumption already around day 5 (1.2 mM). Thereafter, its concentration ranged between 1 and 1.3 mM until day 30.

The temperature-dependent concentration patterns of acetate and propionate were governed by the Gibbs free energies (ΔG). The Δ*G* values indicated that syntrophic acetate oxidation was feasible at 45 °C, but not at 30 °C. By contrast, syntrophic propionate oxidation was permissive at both temperatures, but only after 5 days of incubation at mesophilic temperature. As a consequence, the turnover of acetate and propionate significantly (*P* < 0.001) differed over the 30-day incubation period between 30 and 45 °C (Fig. 4).

The ΔG values for acetoclastic and hydrogenotrophic methanogenesis were mostly negative at both temperatures 30 °C and 45 °C. At mesophilic temperature, CH_4_ production started around day 5 (0.15 kPa) and increased significantly to 24.5 kPa (*P* < 0.001, day 11), 27.5 kPa (day 16), 42.4 kPa (day 23), and 48.5 kPa (day 30). Significant CH_4_ production at thermophilic temperature started around day 11, with greatest production rates between days 23 (9.5 kPa) and 30 (26.0 kPa). The H_2_ partial pressure at 30 °C was significantly (*P* < 0.001) higher until day 11 than at 45 °C. Thereafter, the H_2_ partial pressure had the same nearly undetectable level regardless of temperature (30 °C or 45 °C).

### Syntrophy

The gene encoding formyltetrahydrofolate synthetase (*fhs*, EC 6.3.4.3) has been widely used as a biomarker for acetogenesis [30–33]. It is a unique pathway gene in reductive acetogenesis (acetyl-CoA pathway); however, formyltetrahydrofolate synthetase is also able to catalyze the reverse reaction, oxidizing acetate into H_2_ and CO_2_ [34, 35]. Thus, depending on the thermodynamic conditions, *fhs* transcripts are indicative of either reductive acetogenesis or syntrophic acetate oxidation.

**Fig. 5:**
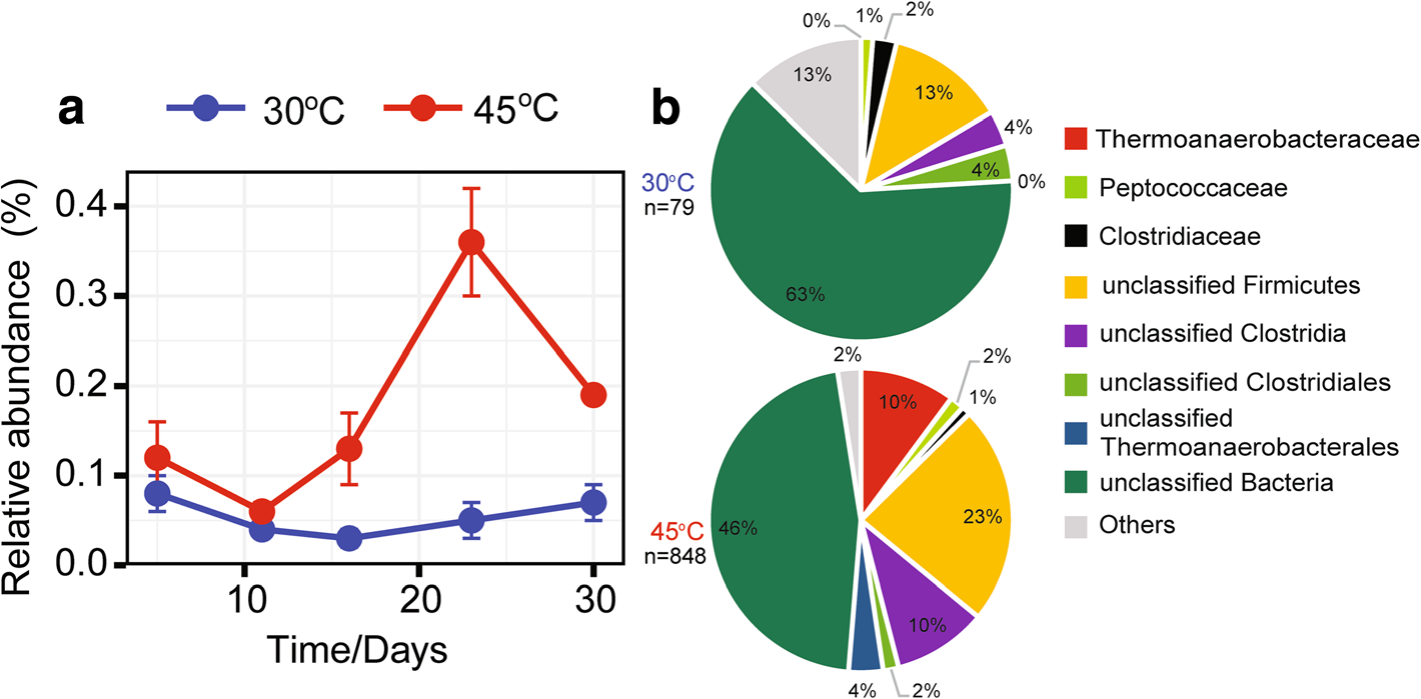
Temporal abundance dynamics of formyltetrahydrofolate synthetase (*fhs*) transcripts at 30 °C and 45 °C (means ± SE, *n* = 3) **(a)** and their taxonomic composition **(b)**. For taxonomic analysis, all *fhs* reads obtained for either 30 °C or 45 °C were combined to a single dataset due to the low number of transcripts obtained for each time point (*n* = total number of *fhs* reads that were taxonomically assigned)

The relative expression level of *fhs* was significantly increased (*P* < 0.001) at 45 °C (926 reads, 0.17% [total mRNA]) relative to 30 °C (83 reads; 0.06% [total mRNA]). The temperature-dependent difference in *fhs* abundance was most significant on day 23. The good correspondence between acetate turnover, Gibbs free energy calculations, and temperature-dependent *fhs* transcript dynamics confirmed that syntrophic acetate oxidation occurred at 45 °C, but not at 30 °C (Figs. 4 and 5).

Due to limited read numbers, we merged the *fhs* transcripts of all samplings to a single dataset for taxonomic identification. The vast majority of *fhs* transcripts at thermophilic temperature could not be assigned on family level, except those affiliated with the *Thermoanaerobacteraceae* (Fig. 5). Their *fhs* transcript abundance significantly increased (*P* < 0.01) with incubation time and showed a peak abundance on day 23 (Fig. 5a). The combined analysis of the rRNA and mRNA dynamics revealed a temporal co-occurrence of a 16S rRNA population assigned on family level to the *Heliobacteriaceae* and the *fhs* transcript pool (Figs. 1c and 5). A closer inspection showed that this rRNA population is comprised of rRNA reads assigned to *Heliobacteriaceae* and “unclassified *Firmicutes*” (31.7% and 68.3% on day 23, respectively). On genus level, the “unclassified *Firmicutes*” rRNA reads were assigned to *Hydrogenispora* with only a single described species (*H. ethanolica*) for which no genome sequence is available yet. This species belongs to the monophyletic *Firmicutes* clade OPB54 (Fig. 1c) that, except for *H. ethanolica*, consists only of environmental 16S rRNA gene sequences. Thus, co-occurrence in the peak abundances of this 16S rRNA-based population and the *fhs* transcript pool let us conclude that in addition to *Thermoanaerobacteraceae*, members of the *Heliobacteriaceae* and, in particular, the *Firmicutes* clade OPB54 contribute to syntrophic acetate oxidation.

Unlike syntrophic acetate oxidation, there is no specific marker gene that is indicative of syntrophic propionate oxidation. Therefore, we extracted the mRNA reads affiliated with two different types of syntrophic propionate oxidation pathways. The transcripts related to these two pathways made approximately 2.5% (30 °C) and 1.0% (45 °C) of total mRNA. The bacterial populations involved in propionate metabolism greatly differed on family level between 30 and 45 °C. At mesophilic temperature, the mRNA profiles were dominated by the *Symbiobacteriaceae* and *Peptococcaceae*. Their mRNA abundances showed opposing temporal dynamics. Most of the transcripts detected at 45 °C were affiliated with *Thermoanaerobacteraceae*, *Ruminococcaceae*, and *Peptococcaceae* (Additional file 1: Figure S6).

### Methanogenesis

**Fig. 6:**
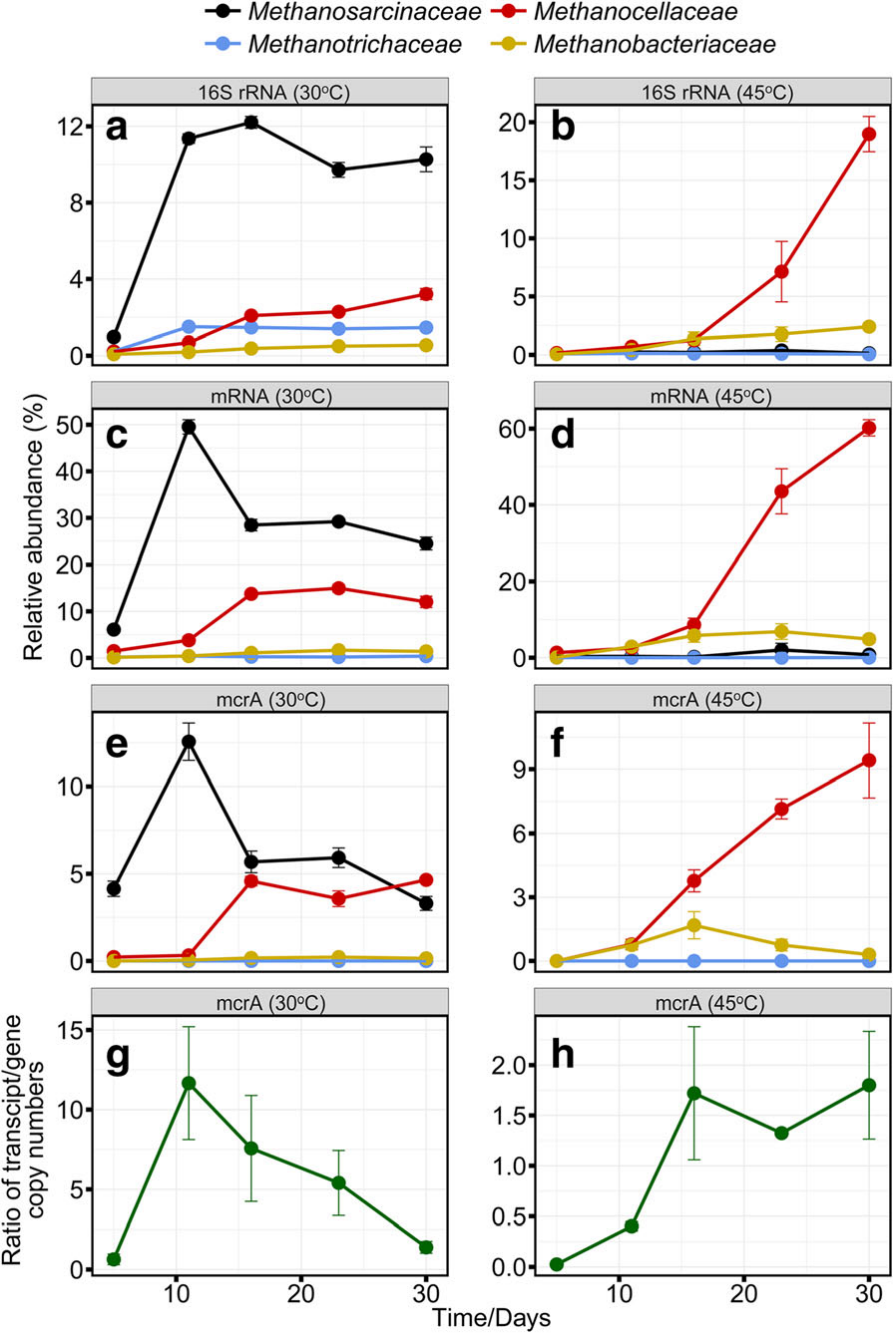
Family-level dynamics of archaeal 16S rRNA (**a, b**), methanogenic mRNA (**c, d**) and *mcrA* transcripts (**e, f**) in metatranscriptomic analysis (means ± SE, *n* = 3) and the temporal dynamics of the *mcrA* transcript/gene ratio (**g, h**) as determined by qPCR (gene) and RT-qPCR (transcripts) (means ± SE, *n* = 3). Results are shown for 30 °C and 45 °C

At mesophilic temperature, acetoclastic *Methanosarcinaceae* dominated the early incubation stage. Their abundance decreased between days 11 and 16. Concomitantly, the abundance of H_2_/CO_2_-utilizing *Methanocellaceae* increased. These opposing abundance dynamics were most evident on mRNA level (Fig. 6 and Additional file 1: Figure S7) and related to a transient decrease in CH_4_ production (Fig. 4). Transcripts of individual methanogen proteins showed the same temporal abundance patterns, including methyl coenzyme M reductase (McrA) (Additional file 1: Figures S7 and S8), acetyl-CoA decarbonylase/synthase complex (ACDS), and 5, 10-methylenetetrahydromethanopterin reductase (Mer)/coenzyme F420 hydrogenase (Frh). The latter two enzyme complexes are characteristic of either acetoclastic (ACDS) or hydrogenotrophic (Mer/Frh) methanogenesis (Additional file 1: Figures S7 and S8).

At thermophilic temperature, the pool of methanogen mRNA was dominated by transcripts indicative of hydrogenotrophic methanogenesis (up to 99.96 ± 0.06%) (Additional file 1: Table S5) and related to the *Methanocellaceae*. Members of the family *Methanobacteriaceae* were also detected, albeit with low abundance. According to their *mcrA* transcript dynamics, the greatest activity of the *Methanobacteriaceae* was around day 16 (Fig. 6f).

**Fig. 7:**
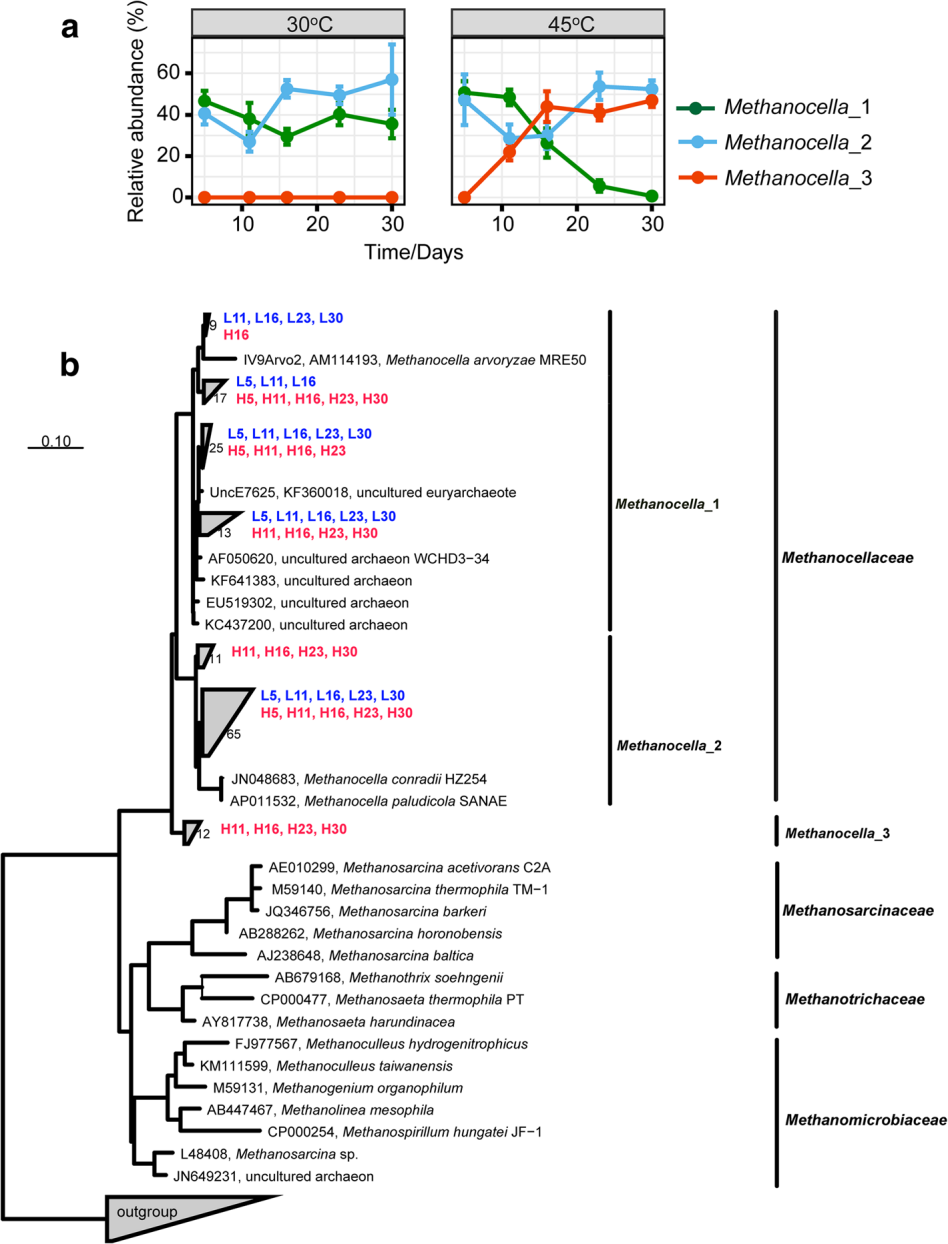
Temporal dynamics of distinct populations within the family *Methanocellaceae*. The analysis is based on nearly full-length 16S rRNA sequences assembled by EMIRGE (means ± SE, *n* = 3) (**a**). Phylogenetic tree recovered by EMIRGE from archaeal 16S rRNA reads of the metatranscriptomic datasets. Ribo-contigs affiliated with the *Methanocellaceae* and assembled in this study are shown in color: L (30 °C), H (45 °C), with numbers representing the incubation time point. The outgroup is defined by bacterial reference sequences (**b**)

Near full-length 16S rRNA sequences of the *Methanocellaceae* clustered into three phylogenetically distinct populations (Fig. 7). *Methanocella*_2 was prevalent at 30 °C and 45 °C; however, temperature had a significant effect on the intrafamily rRNA dynamics of *Methanocella*_1 and *Methanocella*_3. *Methanocella*_1 was prevalent at 30 °C, but not at 45 °C. The *Methanocella*_1 population steadily decreased in abundance from 50.8% (day 5) to a low level on day 30. By contrast, *Methanocella*_3 was undetectable at 30 °C but rapidly increased in abundance from < 0.5% (day 5) to 46.9% (day 16) at 45 °C. Thereafter, the intrafamily rRNA abundance of this yet uncultured *Methanocellaceae* population did not significantly change until day 30 (Fig. 7).

At 30 °C, the dynamics of the *mcrA* transcript to gene ratio corresponded closely with the relative abundance changes of both total mRNA and *mcrA* transcripts from *Methanosarcinaceae* (Fig. 6). At 45 °C, the dynamics of the *mcrA* transcript to gene ratio corresponded well with the relative abundance changes of *mcrA* transcripts from *Methanocellaceae* and, in addition, showed a nearly perfect correspondence to the intrafamily abundance dynamics of the *Methanocella*_3 population (compare Figs. 6h and 7a).

## Discussion

We tested the impact of two contrasting temperatures on the anaerobic food web in rice field soil. The mesophilic temperature (30 °C) relates to the present-day conditions in most rice paddies, while the thermophilic temperature (45 °C) represents a scenario that may occur in various tropical and subtropical rice paddies in the near future due to global warming. Rice straw was added as the major source of carbon in CH_4_ production. The incorporation of rice straw into the soil strongly enhances the emission of CH_4_ from rice fields [14, 36, 37]. Methanogenic activity in paddy soil without rice straw is low [26, 38]. While the following text discusses the differential effects of temperature on the anaerobic food web, a detailed excursus on polymer breakdown, including a critical assessment of the expression of GH transcripts by methanogenic archaea, is made in the Additional file 3.

### Differential temperature effect on the structure and function of the anaerobic food web

The mesophilic and thermophilic food web communities had a similar modular structure consisting each of three complex modules. Corresponding network modules (LM2/HM2; LM3/HM3) had various family-level groups in common. In particular, the affiliation of methanogenic families to either LM2/HM2 (*Methanosarcinaceae*, *Methanotrichaceae*) or LM3/HM3 (*Methanocellaceae*, *Methanobacteriaceae*) suggested that at both temperatures, the food web communities were governed by similar metabolic interactions. A thorough inspection of the LM and HM networks, however, revealed that along the time axis, temperature had a differential effect on the structural and functional continuum in which the food web operates. This is most evidenced by the rRNA dynamics of family-level groups affiliated with each particular module. These taxon-specific dynamics indicated that each module has a particular functional role within the food web. Corresponding modules, however, differ in their functional role. LM2 is characterized by acetoclastic methanogenesis, whereas HM2 is specified by polymer hydrolysis and primary fermentation. Both LM3 and HM3 are characterized by hydrogenotrophic methanogenesis, but CH_4_ production is linked to syntrophic oxidation of different intermediates (propionate [30 °C] vs. acetate [45 °C]). Common to both temperatures is that the first module (LM1, HM1) represents the early incubation stage. Their members showed no co-occurrence with methanogens and, except for *Clostridium* cluster XVIII (LM1) and *Bacillaceae* (HM1), were mostly of minor abundance. These populations may have been members of the starting community or initially proliferated by utilizing alternative electron acceptors, such as Fe(III), nitrate, sulfur, or sulfate. In anoxic paddy soil, alternative electron acceptors are completely used up during the first few days upon flooding [5].

The gene expression dynamics of *Bacteria* and *Archaea* significantly differed between the two temperatures, thereby providing another line of evidence for a differential temperature effect on the anaerobic food web (Fig. 2). At mesophilic temperature, bacterial and archaeal mRNAs were expressed at low but constant level throughout incubation time, with no particular peak abundances. By contrast, their mRNA expression at thermophilic temperature was characterized by strong abundance dynamics. Community-wide transcription showed two distinct activity stages characterized first by polymer hydrolysis and fermentation (days 5–16) and then followed by syntrophic acetate oxidation coupled to hydrogenotrophic methanogenesis (days 16–30). Syntrophy as the predominant process during the later stage is further evidenced by the co-occurrence link between the increasing mRNA expression activities of *Bacteria* and *Archaea*.

Temperature significantly affects polymer breakdown in rice field soil. The strongly differing CAZyme transcript dynamics provide first-time molecular evidence. Previous enzymatic research has shown that at mesophilic temperature, the hydrolytic release of easily degradable polymer substances occurs over the first 30 days of incubation and becomes increasingly the rate-limiting step in microbial activity after 14 days [39, 40]. The occurrence of polymer hydrolysis over the complete 30-day incubation period is well supported by the abundance dynamics of plant polymer degraders (discussed below). Apparently, this process is characterized by a low but constant expression level of CAZyme transcripts. At thermophilic temperature, the expression level of CAZyme transcripts was significantly increased. The GH/CBM transcript abundance peaked between days 11 and 16, in good agreement with the temporal expression dynamics of total bacterial mRNA. Thereafter, the CAZyme transcript level steadily decreased, suggesting that syntrophic acetate oxidizers were not majorly involved in polymer hydrolysis. The temporal separation of polymer breakdown and syntrophic acetate oxidation may have several reasons. On the one hand, it is well established that thermophilic conditions generally result in an increase in polymer degradation rates, which is attributed to increased hydrolytic activity [41]. On the other hand, the metabolic interaction between acetate oxidizers and *Methanocellaceae* may be characterized by growth rates that involve a substantial delay time until critical sizes of syntrophic populations are established, leading to net consumption of acetate.

### Gradual plant polymer breakdown drives acetoclastic methanogenesis and syntrophic propionate oxidation at mesophilic temperature

The gradual decomposition of plant polymers over the complete incubation period of 30 days is corroborated by the persistent presence of family-level groups involved in polymer hydrolysis. These groups belonged to the low-complexity modules LM4, LM5, and LM6 (i.e., *Bacillaceae*, *Clostridiaceae*, *Lachnospiraceae*, *Peptococcaceae*). Other family-level groups were affiliated with LM2 (i.e., *Methanosarcinaceae*, *Ruminococcaceae*) but were also abundantly present during the late incubation stage. On CAZyme transcript level, the *Firmicutes* abundance, however, steadily decreased till day 30. Concomitantly, *Proteobacteria*, *Planctomycetes*, *Bacteroidetes*, and *Ignavibacteriae* increased in abundance (Fig. 3). Relative to their contribution to total bacterial mRNA, non-*Firmicutes* were significantly overrepresented in the GH/CBM transcript pool during the later stage (Fig. 3 and Additional file 1: Figure S4). This is most evident for the phylum *Bacteroidetes* that, after *Firmicutes*, contributed second most to the bacterial GH/CBM transcript pool. *Bacteroidetes* are well-known for their important role in polymer degradation [42, 43]. Conclusively, the degradation of plant polymers at 30 °C involves a complex functional interaction of diverse bacterial taxa, in which the contribution of non-*Firmicutes* populations strongly increased with incubation time (Fig. 3). One may speculate that the abundance increase in non-*Firmicutes* is causally linked to the previous finding that the easily degradable polymer substances become gradually exhausted during the later incubation stage [40].

*Methanosarcinaceae* and, to a lesser extent, *Methanotrichaceae* determine the functional role of LM2. It has been shown that *Ruminococcus* produces increased amounts of acetate in the presence of methanogens. This redirection of the fermentation process may explain the temporal co-occurrence of *Methanosarcinaceae* and *Ruminococcaceae*. Acetoclastic methanogenesis as the dominant process of CH_4_ production during the early stage is well confirmed by the RNA dynamics, with concurrent peak abundances of rRNA, total mRNA, and transcripts encoding particular methanogenic enzymes (MCR, CODH) (Fig. 6 and Additional file 1: Figures S7 and S8) on day 11. The subsequent shift from acetoclastic methanogenesis towards increasing contribution of hydrogenotrophic methanogenesis to total CH_4_ production, however, is clearly more pronounced on mRNA level than on rRNA level, reinforcing the view that mRNA is more responsive to environmental change than rRNA [44]. The reduced but steady level of methanosarcinal mRNA during the later stage relates well to the previous result that polymer hydrolysis becomes increasingly the rate-limiting step in microbial activity after 14 days of incubation [40]. Thus, ongoing acetoclastic methanogenesis is presumably driven by acetate produced through rate-limiting polymer breakdown and syntrophic oxidation of propionate [23]. The *mcrA* gene is present only in one or two copies in methanogen genomes [45, 46], thereby making it possible to relate the temporal dynamics in its transcript to gene ratio to changes in cellular expression level. Thus, it is evident that the significant abundance increase in methanosarcinal *mcrA* transcripts between days 5 and 11 is primarily related to a strong increase in their cellular expression level, in addition to growth effects and the increase in methanogen population size (Fig. 6g). The *mcrA* transcript to gene ratio values are similar to those previously observed for highly active acetoclastic methanogens in peatlands [46] and Chinese paddy soils [47].

LM3 is characterized by syntrophic interaction between propionate oxidizers and H_2_/CO_2_-utilizing *Methanocellaceae*. We identified three bacterial families to be involved in propionate metabolism: *Christensenellaceae* (rRNA level) as well as *Symbiobacteriaceae* and *Peptococcaceae* (mRNA level). *Christensenellaceae* and methanogens significantly co-occur in the gut microbiome of human individuals with low body mass index [48]. The same group of authors also showed that in the gut of mice, elevated levels of propionate and butyrate are linked to the co-occurrence of *Christensenellaceae* and hydrogenotrophic methanogens. In our study, *Christensenellaceae* also showed a significant co-occurrence pattern with the *Methanocellaceae* (Fig. 1); however, their functional role still needs to be elucidated. The two known pathways of propionate degradation are (i) methyl-malonyl-coenzyme A (CoA) pathway and (ii) disproportionation of two propionate molecules to acetate and butyrate followed by syntrophic β-oxidation. The expression of mRNA affiliated with these two pathways by *Christensenellaceae* could not be confirmed.

Members of the *Bacteroidetes* and *Firmicutes* can operate the methyl-malonyl-coenzyme pathway in the reverse direction to produce propionate [49, 50]. Linking propionate turnover with the dynamics of propionate metabolism-related mRNA, it is reasonable to assume that *Symbiobacteriaceae* were primarily involved in propionate production (Fig. 4 and Additional file 1: Figure S6). Members of this family have been detected in paddy soils [51] and shown to be capable of producing propionate [52]. Most likely, *Peptococcaceae* were the major players in syntrophic propionate oxidation (Additional file 1: Figure S6). Their mRNA expression dynamics corresponded well to those of the *Methanocellaceae*. Propionate-oxidizing *Peptococcaceae* were previously detected with high abundance in paddy soil [21, 23] and Arctic peat [33].

### The anaerobic food web at thermophilic temperature is a two-stage process driven by polymer breakdown and syntrophic acetate oxidation

The decomposition of plant polymers only involved members of the *Firmicutes*, which is well supported by their overwhelming contribution to total bacterial mRNA (Fig. 3 and Additional file 1: Figure S4). *Clostridiaceae* and *Ruminococcaceae* were dominantly affiliated with HM2 and the central players in polymer breakdown. These two families contributed most to the expression of key enzymes, such as cellulase, endo-1,4-beta-xylanase, alpha-galactosidase, and beta-galactosidase. Their rRNA dynamics corresponded well with the transcript dynamics of cellulase and, to a lesser extent, endo-1,4-beta-xylanase. The abundance of both transcript types peaked on day 16. Their similar expression dynamics suggest that the degradation of cellulose and xylan is highly interrelated [53, 54].

HM3 was characterized by the co-occurrence of syntrophic acetate oxidizers and *Methanocellaceae * (Fig. 1b, c)*.* The contribution of *Thermoanaerobacteraceae* to acetate oxidation was confirmed by their rRNA-based co-occurrence with *Methanocellaceae* and, in addition, *fhs* transcript analysis (Figs. 1b,c and 5). Several genera collectively contributed to the abundance increase of the *Thermoanaerobacteraceae* in co-occurrence with the *Methanocellaceae* (data not shown). These genera are represented by the thermotolerant *Tepidanaerobacter acetatoxydans* [55] and *Syntrophaceticus schinkii* [56], and the thermophilic *Thermacetogenium phaeum* [20]. Members of these genera are known for their ability to oxidize acetate syntrophically [20, 41, 42].

However, the greatest contribution to syntrophic acetate oxidation was made by unknown or yet uncharacterized populations that had been grouped in *fhs* transcript analysis as “unclassified *Firmicutes*” or even “unclassified *Bacteria*”. The rRNA dynamics of *Heliobacteriaceae* and OPB54 agreed perfectly with the relative *fhs* gene expression dynamics (Figs. 1 and 5). *Heliobacteriaceae* as a possible candidate for contributing to syntrophic acetate oxidation was previously mentioned in a single report [57]. OPB54 is considered to represent a new but undescribed taxon within the *Firmicutes* at the rank of a new order or even class [58]. On rRNA gene level, the abundant presence of OPB54 in methanogenic thermophilic reactors (55 °C) has been observed, but their functional role remained elusive. Given that no genomic information is available for OPB54, its members may be responsible for the expression of a large proportion of *fhs* transcripts categorized as unclassified *Bacteria*, *Firmicutes*, or *Clostridia*.

Hydrogenotrophic methanogenesis was overwhelmingly dominated by *Methanocellaceae*, as confirmed by analysis of total RNA, mRNA, and *mcrA* transcripts. This increase in methanogenic activity was related to a strong proliferation of *Methanocella*_3 (Fig. 7). *Methanocellaceae* are known to have two temperature optima at ~ 41 °C and 50 °C [59], but temperature preference under resource competition has not yet been related to a particular phylogenetic clustering. Our results indicate a relationship between phylogeny and temperature preference, with *Methanocella*_1 populations preferring mesophilic conditions. The strong proliferation of *Methanocella*_3 at 45 °C may suggest that this population represents a new *Methanocella* ecotype with a third previously unknown temperature optimum around 45 °C. *Methanocellaceae* outcompeted *Methanobacteriaceae* during the later incubation period (Fig. 6), possibly due to their ability to lower the partial pressure of hydrogen below the level that can be utilized by *Methanobacterium* spp. [14, 60]. The nearly perfect correspondence between the temporal dynamics of *mcrA* transcript to gene ratio (Fig. 6h) and intrafamily rRNA abundance of *Methanocella*_3 (Fig. 7a) suggests a functional link between the increase in *Methanocella*_3 population size and the *mcrA* expression level of its cells.

## Conclusions

**Fig. 8:**
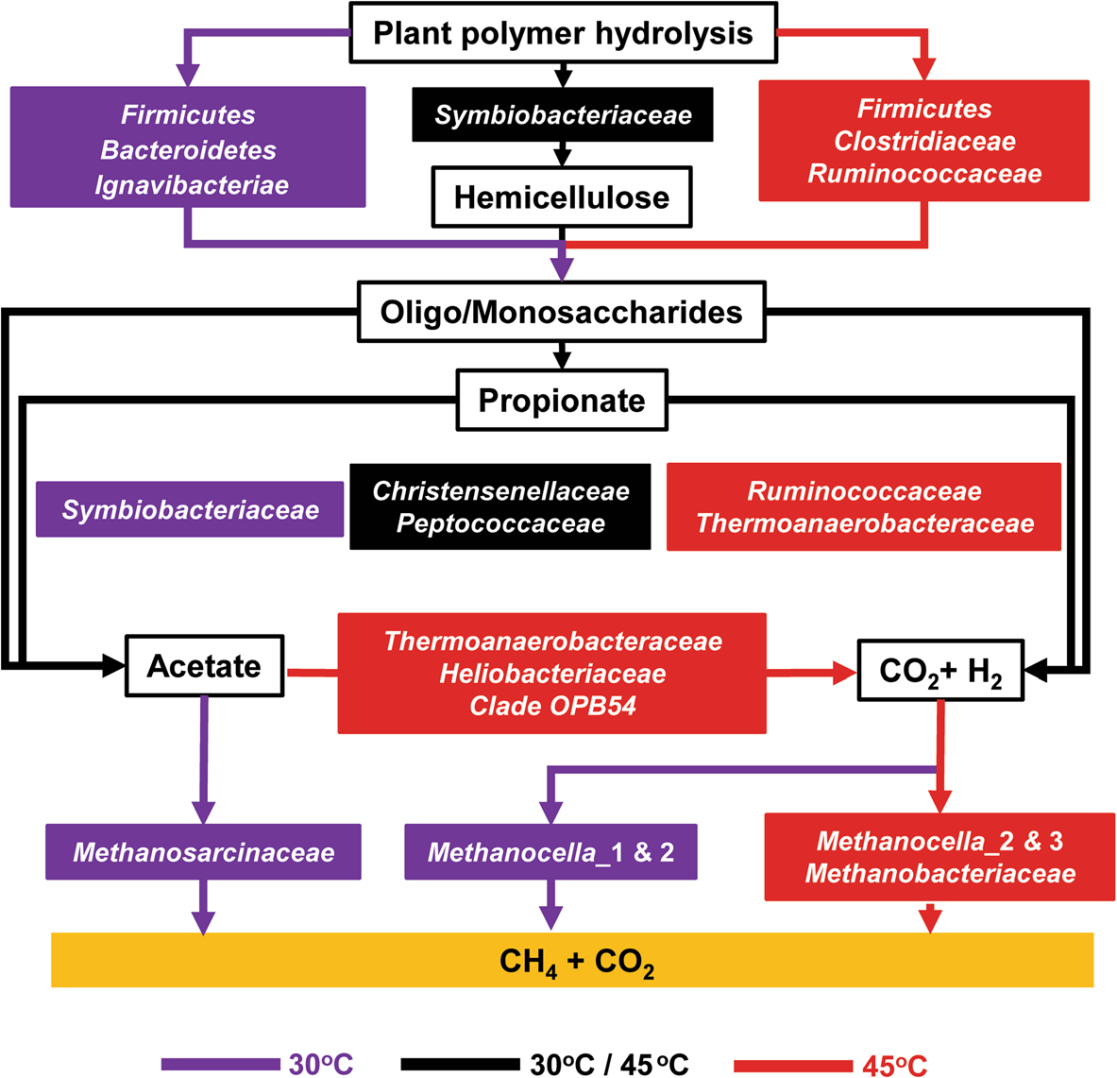
Hypothetical model of the anaerobic food web in rice field soil. The temperature-dependent contribution of particular microbial groups is indicated by different colors. The figure is adapted from Fig. 1 in [4]

The anaerobic food web is driven by the functional interplay of a complex microbial community (Fig. 8). Some taxa were newly identified as potential members of particular functional guilds, such as *Methanosarcinaceae* (polymer hydrolysis), *Christensenellaceae* (propionate metabolism), and *Heliobacteriaceae* and members of clade OPB54 (syntrophic acetate oxidation). Despite the similarities in modular structure and taxonomic composition, temperature and, in consequence, thermodynamic constraints had a significant impact on all functional processes. At both temperatures, food web functionality was directly linked to polymer hydrolysis and the transition from early to late stage. The temperature-induced change in functionality may not only be a realistic near-future scenario for rice paddies but also for methanogenic wetlands in the tropics and subtropics; however, more research is necessary to elucidate the impact of the climate change-induced change in anaerobic food web functionality on CH_4_ emission. In particular, flux measurements in the field will be required to gain accurate estimates whether and how this change in food web functionality will affect the source strength of flooded rice fields and other methanogenic wetlands for atmospheric CH_4_.

## Methods

### Anoxic incubations

Soil was collected from rice fields at the Italian Rice Research Institute (IRRI) in Vercelli, Italy in June 2013. The soil was air-dried and stored as dry lumps at room temperature. Slurry microcosms were set up by filling 125 mL bottles with 40 g dry soil, 0.5 g rice straw (1–2 cm pieces), and 40 mL of autoclaved water. This amount of rice straw is commonly used in paddy soil slurry studies [14, 18, 61]. It corresponds to 37.5 t ha^−1^, i.e., about three times higher than under field conditions [62]. The thoroughly mixed slurries were sealed with butyl rubber stoppers and flushed with N_2_ to establish anoxic conditions. The bottles were then incubated at either 30 °C or 45 °C for 30 days. All bottles were preincubated for 4 days to initiate methanogenic activity [10, 14, 63]. At both temperatures, slurry materials beneath the top layer were collected at days 5, 11, 16, 23, and 30. The samples were immediately shock-frozen using liquid nitrogen and then kept for molecular analysis at − 80 °C. In addition, gas samples (0.1 mL) and liquid samples (0.5 mL) were taken from the same set of slurries for process measurements. All analyses are based on triplicate slurries, destructively sampled at each time point. Acetate concentration and methane formation were monitored as described previously [64]. The same set of slurries was used for chemical (acetate, propionate, and gas measurements) and molecular analyses.

### Calculations of Gibbs free energies

Gibbs free energy (ΔG) of acetoclastic methanogenesis, hydrogenotrophic methanogenesis, syntrophic acetate oxidation, and syntrophic propionate oxidation was calculated based on the concentrations of gases and dissolved compounds in the slurries incubated at either 30 °C or 45 °C [65]. The Gibbs free energy of different pathways was calculated by using the actually measured partial pressures and concentrations according to the Nernst equation [65].

1. Syntrophic acetate oxidation: $\Delta G(T)=\Delta {H}^{{}^{\circ}}-\mathrm{RBT}+\mathrm{RT}\bullet \ln \frac{p{\left({\mathrm{H}}_2\right)}^4\bullet p{\left({\mathrm{CO}}_2\right)}^2}{\left[{\mathrm{Ac}}^{-}\right]\left[{\mathrm{H}}^{+}\right]}$
2. Syntrophic propionate oxidation: $\Delta G(T)=\Delta {H}^{{}^{\circ}}-\mathrm{RBT}+\mathrm{RT}\bullet \ln \frac{\left[{\mathrm{Ac}}^{-}\right]\bullet p\left({\mathrm{CO}}_2\right)\bullet p{\left({\mathrm{H}}_2\right)}^3}{\left[{\mathrm{CH}}_3{\mathrm{CH}}_2{\mathrm{CO}\mathrm{O}}^{-}\right]}$
3. Acetoclastic methanogenesis: $$ \Delta G(T)=\Delta {H}^{{}^{\circ}}-\mathrm{RBT}+\mathrm{RT}\bullet \ln \frac{p\left({\mathrm{CO}}_2\right)p\left({\mathrm{CH}}_4\right)\ }{\left[{\mathrm{Ac}}^{-}\right]\left[{\mathrm{H}}^{+}\right]} $$
4. Hydrogenotrophic methanogenesis: $$ \Delta G(T)=\Delta {H}^{{}^{\circ}}-\mathrm{RBT}+\mathrm{RT}\bullet \ln \frac{p\left({\mathrm{CH}}_4\right)\ }{p{\left({\mathrm{H}}_2\right)}^4p\left({\mathrm{CO}}_2\right)} $$

∆*H*^0^ of each reaction (1–4) is 270.6, 205.1, 17.7, and − 252.9 KJ mol^−1^, respectively. In addition, RB of each reaction (1–4) is 0.723, 0.447, 0.314, and -0.41 KJ mol^-1^ K^-1^ , respectively.

### Nucleic acid extraction

The FastDNA ®SPIN Kit for Soil (MP Biomedicals, Solon, OH) was used for extraction of total DNA. Total RNA was extracted from slurries using a previously described method [66]. Briefly, fresh soil (0.5 g) was mixed with the same volume of glass beads and suspended in 700 μl of TPM buffer (0.5 M Tris pH 7.0, 1.7% polyvinylpyrrolidone, 0.2 M MgCl_2_). The mixture was shaken at 6.0 m s^−1^ for 45 s followed by centrifugation at 20,000×*g* for 4 min. The pellet was resuspended in 700 μl of PBL buffer (0.05 M Tris pH 7.0, 0.05 M Na_2_EDTA, 0.1% SDS *w*/*v*, 6% *v*/*v* phenol) and the lysis procedure was repeated as described above. Supernatants were purified by two-step phase extraction with phenol-chloroform-isoamylalcohol and chloroform-isoamylalcohol. RNA was precipitated with precooled isopropanol and resuspended in 50 μl of TE buffer (10 mM Tris-HCl, 1 mM EDTA [pH 8.0]). RNA extracts were treated with DNase I (Ambion) and purified using the RNA Clean and Concentrator kit (ZymoResearch) according to the manufacturer’s instructions. The integrity of the purified RNA was checked by Bio-Rad Experion™ and RNA HighSens Chips (Bio-Rad).

### RNA-Seq

Thirty samples of total RNA (corresponding to three replicate slurries × 5 time points × 2 temperatures [30 °C, 45 °C]) were subjected to cDNA synthesis using the NEBNext® Ultra™ Directional RNA Library Prep Kit for Illumina® (New England Biolabs, USA) according to the manufacturer’s instructions. cDNA yield and integrity were determined by fluorometry (Qubit®, ThermoFisher, USA) and automated high-resolution electrophoresis (Experion, Bio-Rad, USA). The thirty cDNA libraries were sequenced on an Illumina HiSeq platform in paired-end mode (2 × 250 bp) at the Max Planck Genome Centre Cologne. Illumina RNA-Seq resulted in 133,049,210 reads, ranging from 3,450,734 to 5,841,253 reads per cDNA library (Additional file 1: Table S6).

### Bioinformatic processing of total RNA

Paired-end reads were merged using fastq_mergepairs function (fastq_minovlen 20, fastq_minmergelen 250) in USEARCH7. Quality filtering was performed using the USEARCH7 fastq_filter function with a maximum expected error threshold of 0.5 [67]. Analysis of 16S rRNA and putative mRNA reads was carried out with a customized pipeline as described previously [44].

Briefly, both 16S and 18S rRNA were extracted from total RNA by SortMeRNA 2.0 [68]. A total of 77,665,958 quality-filtered reads was obtained from total RNA, of which 34,458,629 and 1,361,405 reads were derived from 16S rRNA and 18S rRNA, respectively (Additional file 1: Table S6). USEARCH7 and QIIME (version 1.9.1) [69] were used as the overall framework for rRNA-derived reads. OTU clustering was done using the UCLUST algorithm integrated into USEARCH and an OTU threshold of 95% sequence identity. Using a custom PYTHON script, the resulting OTU table was converted to match the particular specifications of QIIME. Taxonomic assignments were done using the RDP classifier [70] implemented in QIIME with SILVA SSU (release 128) as the reference database. Due to the low proportion of eukaryal 18S rRNA, our study focused on bacterial and archaeal 16S rRNA. In particular, fungi were nearly not detectable. They contributed between 0.03 and 0.3% to total SSU rRNA reads (Additional file 1: Tables S7 and S8).

For mRNA analysis, reads mapping to rRNA and non-coding small RNA were filtered by SortMeRNA 2.0, using SILVA (release 128) and RFAM as reference databases. The remaining reads were considered putative mRNA. We identified only a low proportion of mRNA reads (0.39–3.20%) to be derived from *Eukaryota* (Additional file 1: Table S9). Like for SSU rRNA, we therefore extracted only bacterial and archaeal mRNA reads through MEGAN6 Ultimate Edition [71] for downstream analysis. These were a total of 2,245,249 quality-filtered reads (Additional file 1: Table S10). Taxonomic assignment and functional annotation of the mRNA reads were carried out using the UBLAST algorithm implemented in USEARCH7 applying an *e* value cutoff of 1e-5, maxhits 50, and maxaccepts 50 for database searches against NCBI’s non-redundant protein database (release 2017). MEGAN6 Ultimate Edition was used for parsing and downstream analysis of the UBLAST output. Taxonomy assignments were made by the “lowest common ancestor” (LCA) method using the following parameters: minimum support 1, minimum bit score 150, top percent 10 [72]. A total of 1,242,931 reads had a taxonomic hit in the NCBI’s non-redundant protein database (Additional file 1: Table S10). Taxonomically assigned mRNA was functionally annotated according to SEED subsystems and KEGG categories (release 2017). For in-depth sub-transcriptome analysis, mRNA reads related to particular functional genes and pathways were extracted from MEGAN6 and blasted against NCBI’s non-redundant protein database. UBLAST searches were carried out with an *e* value threshold of 1e-10, followed by the analysis in MEGAN6 Ultimate Edition.

### Assembly of full-length *Methanocellaceae* 16S rRNA sequences

All the Illumina reads affiliated with 16S rRNA of the *Methanocellaceae* were extracted by Python scripts and used to reconstruct near full-length 16S rRNA sequences via 60 iterations in EMIRGE. The abundance estimate of assembled reads per 16S rRNA sequence is NormPrior, which is a length-normalized estimate determined by the EMIRGE algorithm [73]. Each 16S rRNA sequence assembled by EMIRGE is the result of Illumina reads > 97% similarity. Assemblies were done separately for each sequence dataset (= replicate slurry) rather than pooling the datasets together. Totally, 152 full-length 16S rRNA sequences were produced across all replicate samples. Phylogenetic analysis of assembled 16S rRNA was done with ARB software (http://www.arb-home.de/; version 2016; [74]) as previously described [14]. Sequences were aligned using SINA webaligner (https://www.arb-silva.de/aligner/) and imported into the SILVA database (nr99 (release 128)) obtained from the SILVA homepage [75]. Then, the alignment was checked and corrected manually by ARB_EDIT. Phylogenetic placement was performed relative to euryarchaeal reference sequences. A 16S rRNA-based tree was constructed for sequences having a length > 1200 bp. All 152 nearly full-length *Methanocellaceae* 16S rRNA sequences were added to this tree using the ARB parsimony tool, which allows the addition of sequences to phylogenetic trees without changing global tree topologies [76].

### Analysis of carbohydrate-active enzymes (CAZymes)

CAZyme-affiliated mRNA reads were identified by querying total mRNA datasets against dbCAN, a database for carbohydrate-active enzyme annotation (release 2017) [77]. Database search was done with DIAMOND [78] in blastx mode applying the default *e* value cutoff of 1e-3. Functional CAZyme modules (e.g., cellulose degradation) were defined by grouping related enzymatic functions based on their enzyme commission numbers. A mapping file for functional annotation was created using all available entries in dbCAN. The mapping file was stored as indexed sqlite database object and queried using a custom Python script. The resulting annotations are based on matching dbCAN tophits for mRNA reads queried against the formatted mapping file and defined CAZyme modules.

Subsets of mRNA reads linked to CAZyme functions of interest were obtained using custom Python scripts. These subsets were taxonomically assigned by applying the strategy outlined by Menzel and colleagues [79], which relies on identifying maximum exact matches for query sequences in reference databases using the backward search algorithm implemented in the Burrows-Wheeler transform [80, 81]. Prokaryotic genomes from NCBI RefSeq and NCBI nr were used as reference databases.

### qPCR and RT-qPCR of *mcrA*

Numbers of genes and transcripts encoding methyl coenzyme reductase A (*mcrA*) were quantified with primer set mlas-mod-f/mcrA-rev-r [82]. Total RNA was randomly reverse transcribed using the GoScript Reverse Transcription System (Promega, Mannheim, Germany). Standard curves were constructed using PCR amplicons (*mcrA* genes/transcripts, calibration range from 10 to 10^8^ copies). The analyses were performed using SYBR Green-based assays [44]. qPCR was carried out using a CFX Connect Real-Time PCR detection system (Bio-Rad). Like for RNA-Seq, qPCR and RT-qPCR of *mcrA* were each performed from 30 samples. All reactions were carried out in three technical replicates. The PCR efficiency was 89% (*R*^2^ > 0.99).

### Statistical analyses

Non-metric multidimensional scaling (NMDS) plots were generated in the R (version 3.5.0) software (R Development Core Team, 2014) using the vegan package (version 2.4.4) [83] with the metaMDS function and Bray–Curtis similarity matrices on square-root transformed data. Analyses of Similarity (ANOSIM) of Bray–Curtis similarity were performed to test whether community differences between the (i) two temperatures and (ii) incubation time points in the NMDS ordinations are statistically significant [84].

Co-occurrence networks were constructed for 30 °C and 45 °C using pipelines developed previously [85]. These were based on time-dependent changes in taxon-specific rRNA abundances. Ribosomal RNA reads were clustered into OTUs at 95% sequence identity and all the OTUs assigned on family level were retained for further analysis. Family-level groups with less than 0.5‰ of total reads were discarded. Spearman correlation coefficient 0.6 ≤ *ρ* ≤0.9 and false discovery rate (FDR) corrected *P* value ≤ 0.01 were used for co-occurrence network construction. The number of nodes and edges, average path length, and networks were visualized by Gephi software [86].

## Additional files

Additional file 1: This document contains supporting** Figures S1-S8.** and **Tables S1-S10.** The file is published online as Supporting Information. (PDF 1430 kb)

Additional file 2: Corroborating GH expression by methanogens through blasting their genome sequences (download from NCBI database) against dbCAN database. (XLSX 378 kb)

Additional file 3: Detailed excursus on anaerobic polymer breakdown and the temperature-dependent expression of CAZymes. (PDF 87 kb)
