# Supplementary material for: Metatranscriptomics reveals a differential temperature effect on the structural and functional organization of the anaerobic food web in rice field soil
Source: Microbiome. 2018 Sep 19;6:169. doi: 10.1186/s40168-018-0546-9 (PMC6147125; doi:10.1186/s40168-018-0546-9)
Supplement: Supplementary file 1 — This document contains supporting Figures S1-S8. and Tables S1-S10. The file is published online as Supporting Information. (PDF 1430 kb) [file 40168_2018_546_MOESM1_ESM.pdf]

Metatranscriptomics reveals a differential temperature effect on the structural and functional organization of the anaerobic food web in rice field soil

Jingjing Peng[1], Carl-Eric Wegner[2], Qicheng Bei[1], Pengfei Liu[1], Werner Liesack[1]

Supporting information:

Supplementary Figures 1-8, Supplementary Tables 1-10

Figure S1. 16S rRNA dynamics of family-level groups affiliated with the different low-temperature (LM) modules (see co-occurrence network analysis for further details, Figure 1). Representative taxa shown in Figure 1 are bold-marked.

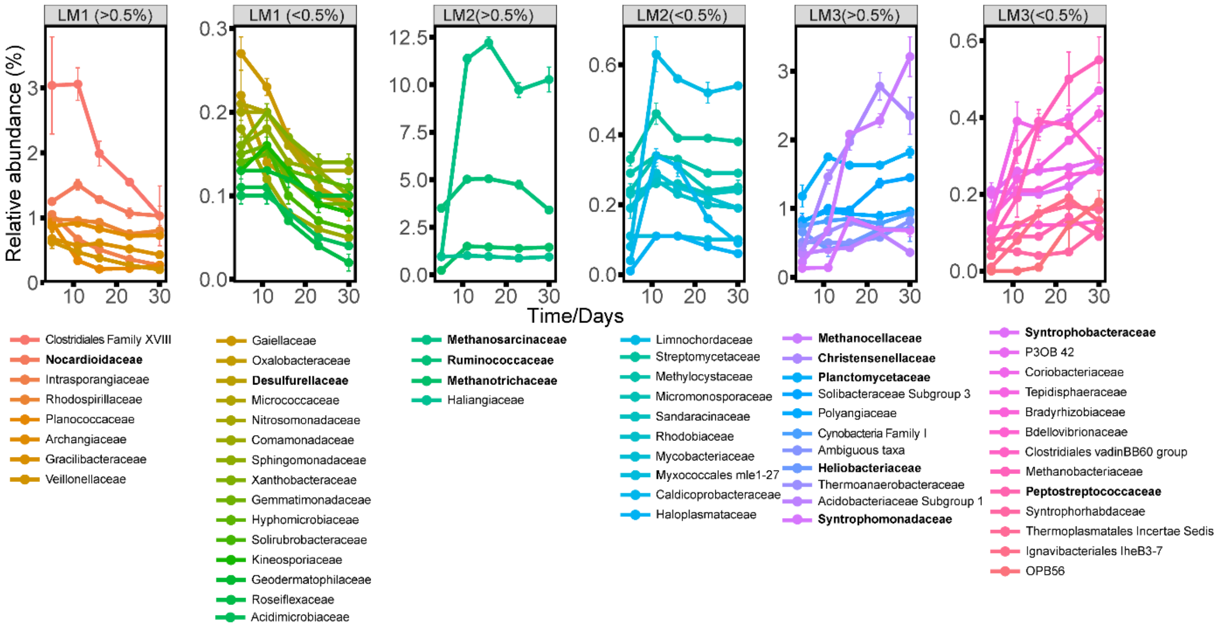

Figure S2. 16S rRNA dynamics of family-level groups affiliated with the different high-temperature (HM) modules (see co-occurrence network analysis for further details, Figure 1). Representative taxa shown in Figure 1 are bold-marked.

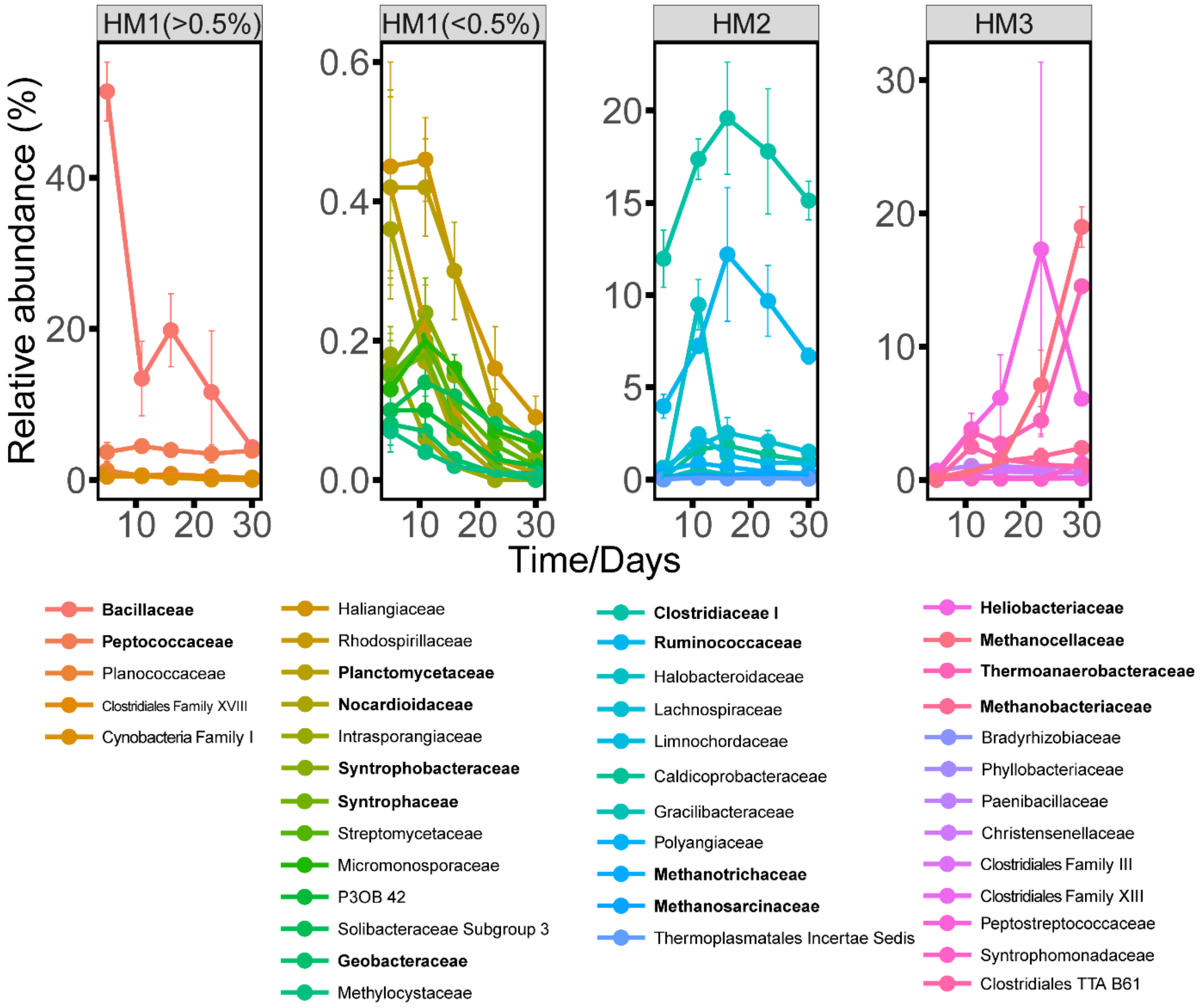

Figure S3. NMDS plots based on Bray-Curtis similarity matrices on 16S rRNA (**a**: family; **b**: phylum) and mRNA (**c**: family; **d**: phylum) levels at 30°C and 45°C.

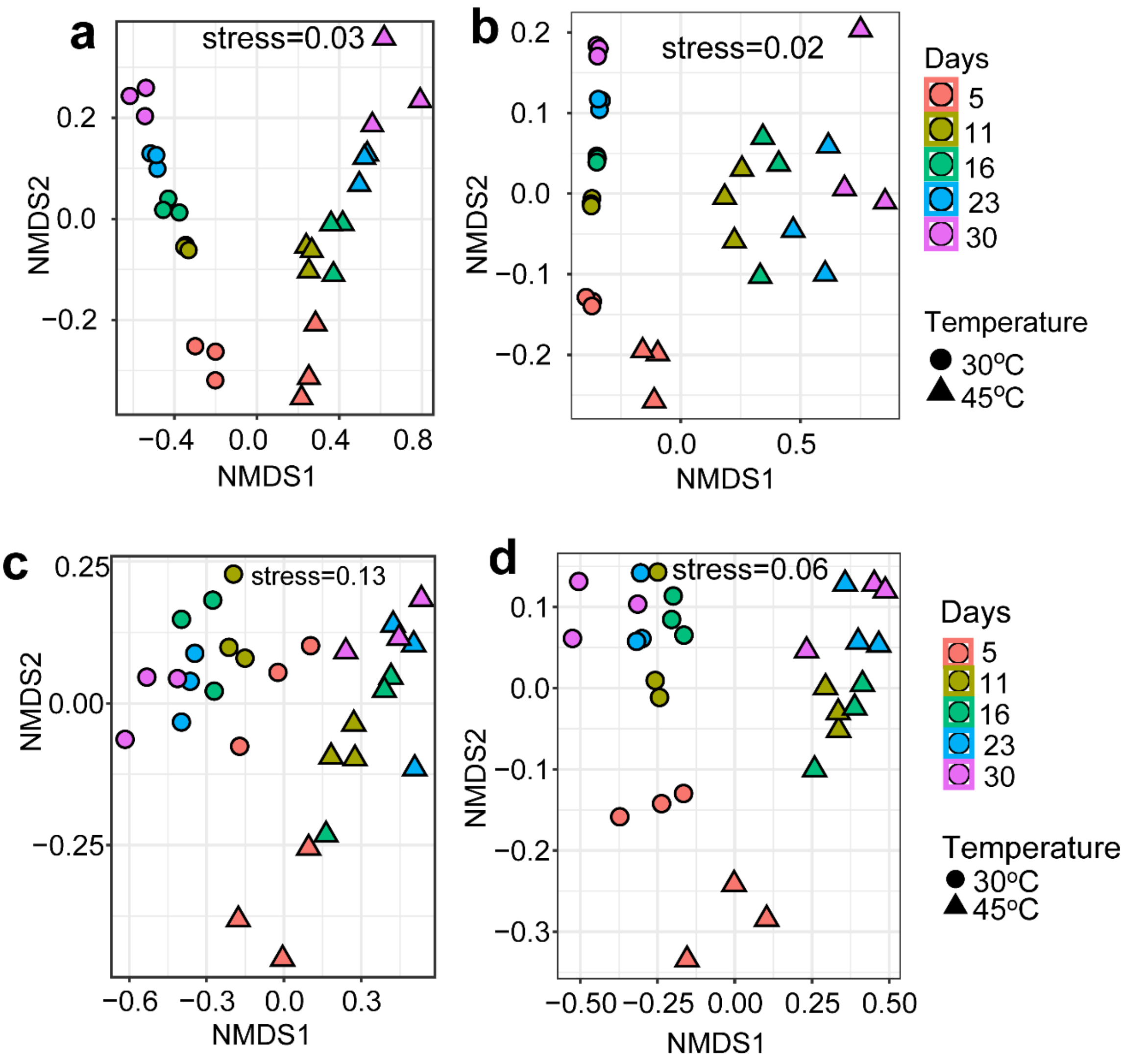

Figure S4. Taxonomic composition of metatranscriptomic data sets on 16S rRNA (**a**: phylum; **c**: family) and mRNA (**b**: phylum; **d**: family) (>1%) levels (means ± SE, n=3).

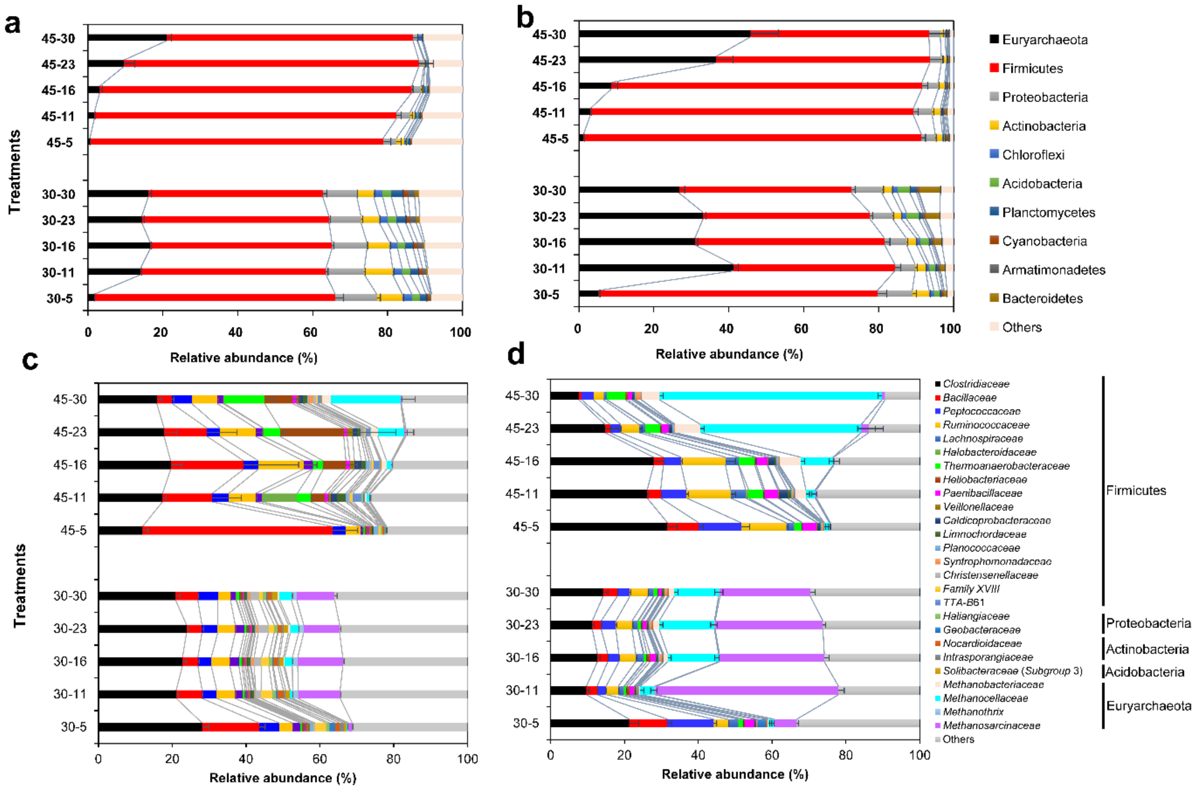

Figure S5. Family-level assignment of GH/CBM transcripts involved in the degradation of cellulose, xylan, other hemicelluloses, and chitin. Taxonomic assignments were done by homology-based assignment against NCBI/RefSeq using diamond and megan with its implemented lowest-common ancestor algorithm (**a**), and by applying the backwards search algorithm implemented in the Burrows-Wheeler-Transform for exact substring matching as the basis for taxonomic assignment of transcripts to degradation pathways (**b**) and key enzymes (cellulase, endo-1,4-beta-xylanase, alpha- and beta-galactosidases) in polymer breakdown (**c**). A detailed discussion of CAZymes involved in polymer breakdown is given in the Additional File 3, including a critical assessment of the taxonomic assignments of GH/CBM transcripts to methanogenic archaea.

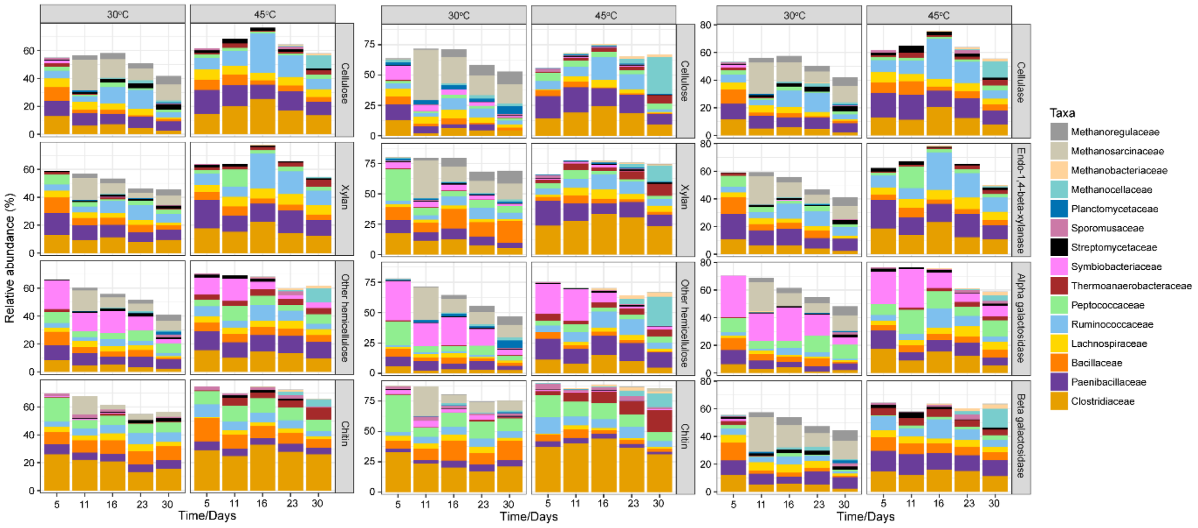

Figure S6. Abundance dynamics of mRNA affiliated with the KEGG category 'propionate metabolism' at 30°C and 45°C (upper panel), and the family-level mRNA dynamics (lower panels). The relative transcript abundances in family-level analysis relate to total mRNA reads affiliated with propionate metabolism (upper panel). Note that mRNA transcripts from replicate slurries (n = 3) were merged due to the low number of transcripts obtained for each sampling time point.

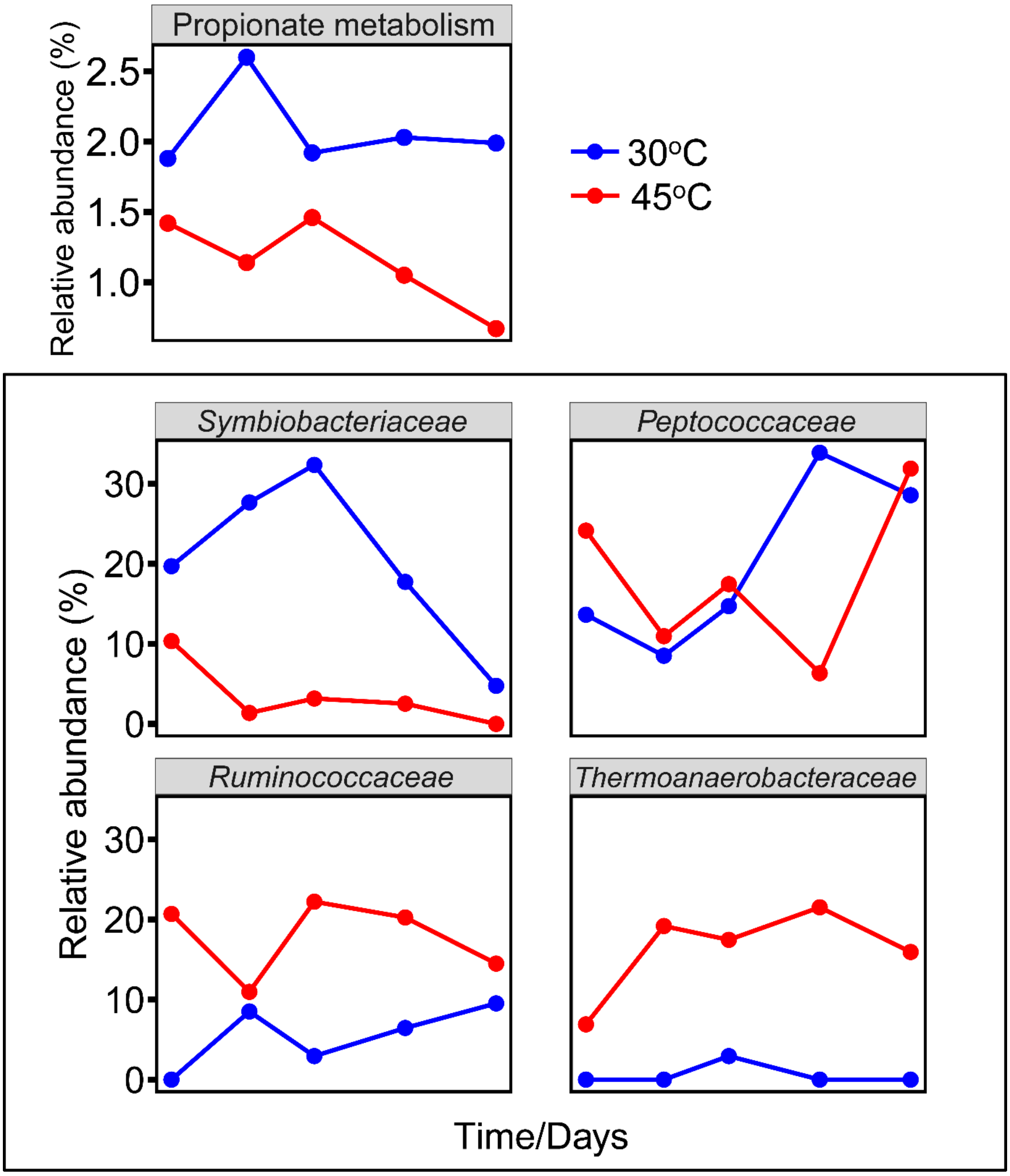

Figure S7. Transcript dynamics of individual genes involved in acetoclastic (ACE) or hydrogenotrophic (HYD) methanogenesis at 30°C and 45°C, according to the KEGG category ‘methane metabolism’. Acetoclastic methanogenesis: mcrA, Methyl coenzyme M reductase; ACDS, acetyl-CoA decarbonylase/synthase complex; Ack, acetate kinase; Acs, acetyl-CoA synthetase; Pta, phosphate acetyltransferase. Hydrogenotrophic methanogenesis: Frh, coenzyme F420 hydrogenase; Mer, 5-10-methylenetetrahydromethanopterin reductase; Fwd, formylmethanofuran dehydrogenase; Ftr, formylmethanofuran dehydrogenase; Mch, methenyltetrahydromethanopterin cyclohydrolase; Mtd, methylenetetrahydromethanopterin dehydrogenase.

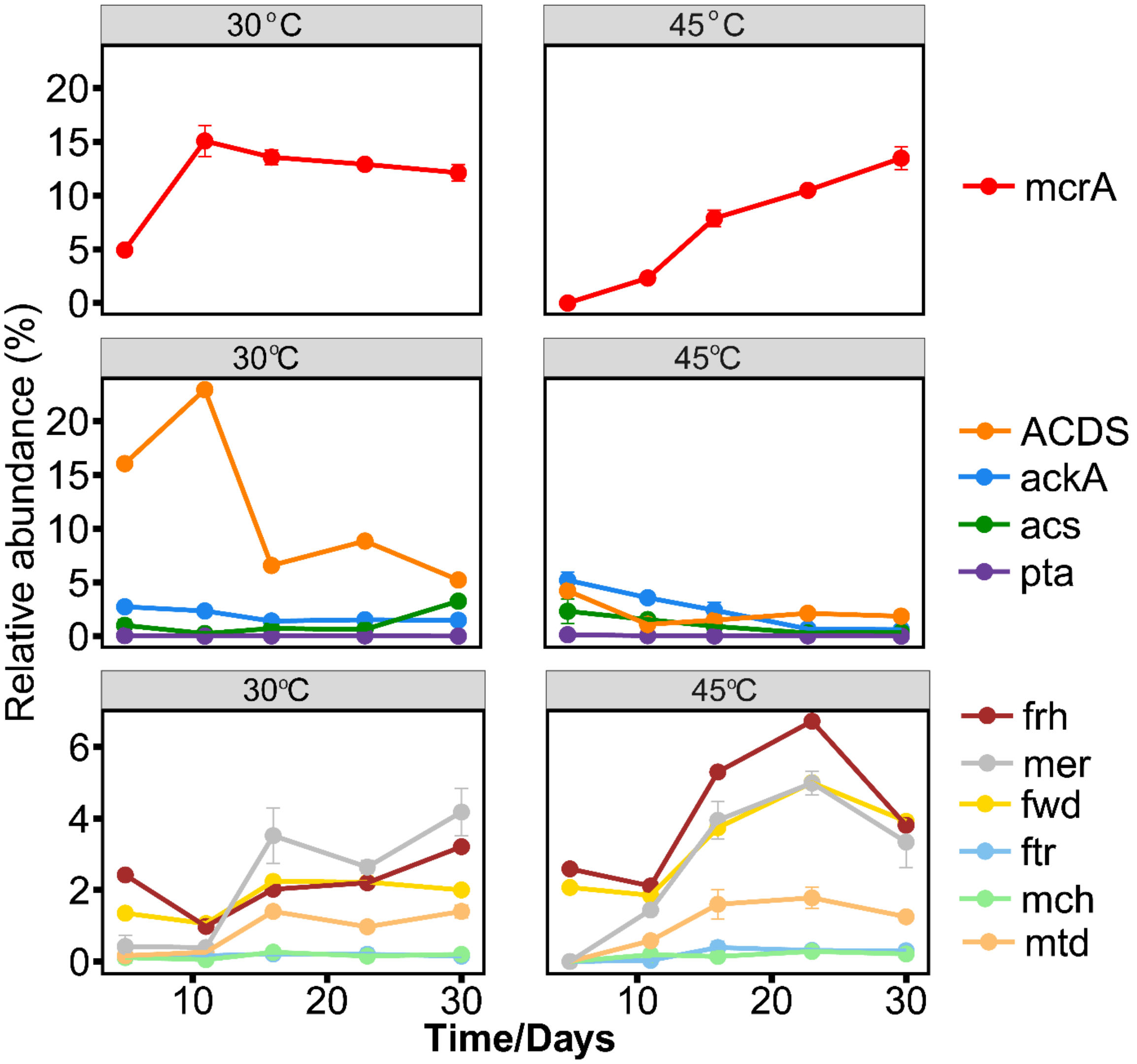

Figure S8. Abundance dynamics of transcripts encoding key functions (y-axis) in the KEGG category 'methane metabolism' in relation to different incubation time points at 30°C and 45°C (x-axis). The size of the circles indicates the relative transcript abundances of key enzymes in the mRNA datasets affiliated with the methane metabolism for *Methanosarcinaceae* (red; 30°C) and *Methanocellaceae* (blue; 45°C).

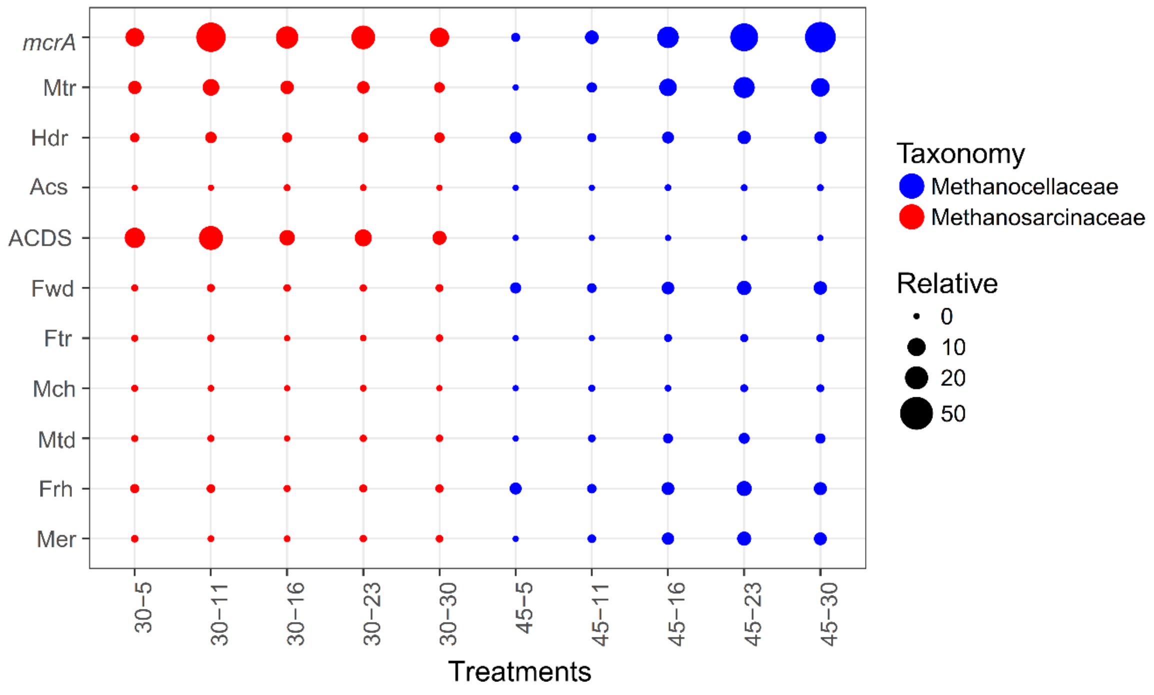

Table S1. Nodes information of 16S rRNA family-based network constructed for 30°C.

Nodes ID	Class	Family	Module	Maximum relative abundance	Degree	Pageranks	Clustering	Triangles
L1	Acidimicrobiia	Acidimicrobiaceae	LM1	0.1	9	0.01	0.50	18
L2	Actinobacteria	Geodermatophilaceae	LM1	0.13	19	0.02	0.49	84
L3	Actinobacteria	Intrasporangiaceae	LM1	1.05	11	0.01	0.75	41
L4	Actinobacteria	Kineosporiaceae	LM1	0.16	20	0.02	0.45	86
L5	Actinobacteria	Micrococcaceae	LM1	0.21	11	0.01	0.76	42
L6	Actinobacteria	**Nocardioidaceae**	LM1	1.28	19	0.02	0.56	95
L7	Alphaproteobacteria	Hyphomicrobiaceae	LM1	0.15	20	0.02	0.59	112
L8	Alphaproteobacteria	Rhodospirillaceae	LM1	0.98	18	0.02	0.61	93
L9	Alphaproteobacteria	Sphingomonadaceae	LM1	0.18	14	0.02	0.73	66
L10	Alphaproteobacteria	Xanthobacteraceae	LM1	0.2	17	0.02	0.57	78
L11	Bacilli	Planococcaceae	LM1	0.93	10	0.01	0.22	10
L12	Betaproteobacteria	Comamonadaceae	LM1	0.12	12	0.01	0.59	39
L13	Betaproteobacteria	Nitrosomonadaceae	LM1	0.2	10	0.01	0.60	27
L14	Betaproteobacteria	Oxalobacteraceae	LM1	0.22	12	0.01	0.73	48
L15	Chloroflexia	Roseiflexaceae	LM1	0.11	8	0.01	0.64	18
L16	Clostridia	Family.XVIII	LM1	3.06	15	0.02	0.73	77
L17	Clostridia	Gracilibacteraceae	LM1	0.68	2	0.00	0.00	0
L18	Deltaproteobacteria	Archangiaceae	LM1	0.92	21	0.02	0.48	100
L19	Deltaproteobacteria	**Desulfurellaceae**	LM1	0.21	14	0.02	0.57	52
L20	Gemmatimonadetes	Gemmatimonadaceae	LM1	0.16	17	0.02	0.66	90
L21	Negativicutes	Veillonellaceae	LM1	0.63	9	0.01	0.36	13
L22	Thermoleophilia	Gaiellaceae	LM1	0.27	12	0.01	0.62	41
L23	Thermoleophilia	Solirubrobacteraceae	LM1	0.16	11	0.01	0.60	33
L24	Actinobacteria	Micromonosporaceae	LM2	0.34	9	0.01	0.36	13
L25	Actinobacteria	Mycobacteriaceae	LM2	0.29	8	0.01	0.43	12
L26	Actinobacteria	Streptomycetaceae	LM2	0.46	8	0.01	0.57	16
L27	Alphaproteobacteria	Methylocystaceae	LM2	0.34	7	0.01	0.67	14
L28	Alphaproteobacteria	Rhodobiaceae	LM2	0.27	10	0.02	0.47	21
L29	Clostridia	Caldicoprobacteraceae	LM2	0.34	7	0.01	0.67	14
L30	Clostridia	**Ruminococcaceae**	LM2	5.05	8	0.01	0.64	18
L31	Deltaproteobacteria	Haliangiaceae	LM2	1.01	4	0.01	0.83	5
L32	Deltaproteobacteria	mle1.27	LM2	0.11	6	0.01	0.60	9
L33	Deltaproteobacteria	Sandaracinaceae	LM2	0.26	7	0.01	0.33	7
L34	Limnochordia	Limnochordaceae	LM2	0.63	9	0.02	0.53	19
L35	Methanomicrobia	**Methanosarcinaceae**	LM2	12.21	7	0.01	0.76	16
L36	Methanomicrobia	**Methanotrichaceae**	LM2	1.5	1	0.00	0.00	0
L37	Mollicutes	Haloplasmataceae	LM2	0.11	8	0.01	0.79	22
L38	Acidobacteria	Acidobacteriaceae..Subgroup.1.	LM3	0.64	4	0.01	0.83	5
L39	Alphaproteobacteria	Bradyrhizobiaceae	LM3	0.26	13	0.02	0.51	40
L40	Chlorobia	OPB56	LM3	0.18	9	0.02	0.75	27
L41	Clostridia	**Christensenellaceae**	LM3	2.78	9	0.02	0.53	19
L42	Clostridia	Clostridiales.vadinBB60.group	LM3	0.39	5	0.01	0.60	6
L43	Clostridia	**Peptostreptococcaceae**	LM3	0.14	6	0.01	0.73	11
L44	Clostridia	**Syntrophomonadaceae**	LM3	0.86	4	0.01	0.50	3
L45	Clostridia	Heliobacteriaceae	LM3	0.7	1	0.00	0.00	0
L46	Clostridia	**Thermoanaerobacteraceae**	LM3	0.8	2	0.01	1.00	1
L47	Coriobacteriia	Coriobacteriaceae	LM3	0.47	12	0.02	0.52	34
L48	Cyanobacteria	FamilyI	LM3	0.93	7	0.02	0.57	12
L49	Deltaproteobacteria	P3OB.42	LM3	0.29	13	0.02	0.56	44
L50	Deltaproteobacteria	**Syntrophobacteraceae**	LM3	0.28	8	0.02	0.61	17
L51	Deltaproteobacteria	Bdellovibrionaceae	LM3	0.13	3	0.01	0.67	2
L52	Deltaproteobacteria	Polyangiaceae	LM3	0.96	7	0.01	0.62	13
L53	Deltaproteobacteria	**Syntrophorhabdaceae**	LM3	0.11	2	0.01	1.00	1
L54	Ignavibacteria	IheB3.7	LM3	0.19	11	0.02	0.40	22
L55	Methanobacteria	**Methanobacteriaceae**	LM3	0.55	8	0.02	0.57	16
L56	Methanomicrobia	**Methanocellaceae**	LM3	3.21	10	0.02	0.51	23
L57	OPB35 soil group	Ambiguous_taxa	LM3	0.82	11	0.02	0.51	28
L58	Phycisphaerae	Tepidisphaeraceae	LM3	0.41	8	0.01	0.68	19
L59	Planctomycetacia	Planctomycetaceae	LM3	1.82	7	0.01	0.76	16
L60	Solibacteres	Solibacteraceae..Subgroup.3.	LM3	1.45	8	0.01	0.68	19
L61	Thermoplasmata	Thermoplasmatales.Incertae.Sedis	LM3	0.17	3	0.01	1.00	3
L62	Anaerolineae	Anaerolineaceae	LM4	0.58	4	0.02	0.00	0
L63	Bacilli	Bacillaceae	LM4	15.4	1	0.01	0.00	0
L64	Bacilli	Paenibacillaceae	LM4	0.66	1	0.01	0.00	0
L65	Clostridia	Peptococcaceae	LM4	5.51	1	0.01	0.00	0
L66	Deltaproteobacteria	Geobacteraceae	LM4	1.56	2	0.01	0.00	0
L67	Clostridia	Clostridiaceae.1	LM5	28.11	1	0.01	0.00	0
L68	Clostridia	Family.XIII	LM5	0.3	1	0.01	0.00	0
L69	Clostridia	Family.XI	LM6	0.33	1	0.01	0.00	0
L70	Clostridia	Lachnospiraceae	LM6	2.43	1	0.01	0.00	0

Table S2. Nodes information of 16S rRNA family-based network constructed for 45°C.

Nodes ID	Class	Family	Module	Maximum relative abundance	Degree	Pageranks	Clustering	Triangles
H1	Acidobacteria/Solibacteres	Solibacteraceae..Subgroup.3.	HM1	0.89	9	0.03	0.25	9
H2	Actinobacteria	Intrasporangiaceae	HM1	0.18	7	0.03	0.05	1
H3	Actinobacteria	Micromonosporaceae	HM1	0.2	7	0.03	0.24	5
H4	Actinobacteria	**Nocardioidaceae**	HM1	0.36	6	0.02	0.00	0
H5	Actinobacteria	Streptomycetaceae	HM1	0.2	8	0.03	0.14	4
H6	Alphaproteobacteria	Methylocystaceae	HM1	0.07	7	0.03	0.10	2
H7	Alphaproteobacteria	Rhodospirillaceae	HM1	0.42	5	0.02	0.10	1
H8	Bacilli	Bacillaceae	HM1	15.4	6	0.03	0.00	0
H9	Bacilli	Planococcaceae	HM1	1.28	5	0.02	0.00	0
H10	Clostridia	Family.XVIII	HM1	0.63	9	0.04	0.06	2
H11	Clostridia	**Peptococcaceae**	HM1	4.49	3	0.01	0.33	1
H12	Cyanobacteria	FamilyI	HM1	0.51	1	0.01	0.00	0
H13	Deltaproteobacteria	**Geobacteraceae**	HM1	0.08	6	0.02	0.00	0
H14	Deltaproteobacteria	Haliangiaceae	HM1	0.46	1	0.01	0.00	0
H15	Deltaproteobacteria	P3OB.42	HM1	0.1	6	0.02	0.27	4
H16	Deltaproteobacteria	**Syntrophaceae**	HM1	0.18	1	0.01	0.00	0
H17	Deltaproteobacteria	**Syntrophobacteraceae**	HM1	0.24	7	0.03	0.00	0
H18	Planctomycetacia	**Planctomycetaceae**	HM1	0.42	3	0.01	0.00	0
H19	Clostridia	Caldicoprobacteraceae	HM2	0.34	17	0.05	0.34	46
H20	Clostridia	Clostridiaceae.1	HM2	28.11	11	0.03	0.33	18
H21	Clostridia	Gracilibacteraceae	HM2	0.54	9	0.03	0.58	21
H22	Clostridia	Halobacteroidaceae	HM2	9.48	7	0.02	0.76	16
H23	Clostridia	Lachnospiraceae	HM2	2.53	9	0.03	0.69	25
H24	Clostridia	Limnochordaceae	HM2	2.45	9	0.03	0.53	19
H25	Clostridia	**Ruminococcaceae**	HM2	12.2	7	0.02	0.43	9
H26	Deltaproteobacteria	Polyangiaceae	HM2	0.89	8	0.02	0.36	10
H27	Methanomicrobia	**Methanotrichaceae**	HM2	0.13	11	0.03	0.29	16
H28	Methanomicrobia	**Methanosarcinaceae**	HM2	0.38	3	0.01	0.67	2
H29	Thermoplasmata	Thermoplasmatales.Incertae.Sedis	HM2	0.09	7	0.02	0.71	15
H30	Alphaproteobacteria	Bradyrhizobiaceae	HM3	0.15	9	0.03	0.47	17
H31	Alphaproteobacteria	Phyllobacteriaceae	HM3	0.21	10	0.03	0.56	25
H32	Bacilli	Paenibacillaceae	HM3	1.05	11	0.03	0.45	25
H33	Clostridia	**Christensenellaceae**	HM3	0.64	18	0.05	0.37	57
H34	Clostridia	Family.III	HM3	0.97	8	0.02	0.46	13
H35	Clostridia	Family.XIII	HM3	0.17	13	0.03	0.50	39
H36	Clostridia	**Heliobacteriaceae**	HM3	17.29	2	0.01	1.00	1
H37	Clostridia	**Peptostreptococcaceae**	HM3	0.15	8	0.02	0.71	20
H38	Clostridia	**Syntrophomonadaceae**	HM3	1.23	10	0.03	0.67	30
H39	Clostridia	**Thermoanaerobacteraceae**	HM3	14.53	9	0.03	0.64	23
H40	Clostridia	TTA.B61	HM3	2.48	7	0.02	0.81	17
H41	Methanobacteria	**Methanobacteriaceae**	HM3	2.39	5	0.02	0.60	6
H42	Methanomicrobia	**Methanocellaceae**	HM3	18.98	5	0.02	0.80	8

Table S3. Mean numbers and percentages (n = 3) of CAZyme transcripts that could be assigned on phylum and family levels at five sampling time points and two temperatures (30°C vs. 45°C).

	**Cellulose**			**Xylan**			**Other hemicelluloses**			**Chitin**		
	Number	Phylum (%)	Family (%)	Number	Phylum (%)	Family (%)	Number	Phylum (%)	Family (%)	Number	Phylum (%)	Family (%)
**30-5**	176	96.03	77.73	335	97.40	81.15	400	96.93	80	707	98.13	85.02
**30-11**	237	97.09	77.56	412	96.55	76.96	448	96.33	76.58	505	97.13	81.56
**30-16**	185	95.02	74.85	359	96.44	74.38	364	96.60	70.57	412	96.57	76.89
**30-23**	260	94.12	66.43	469	94.71	69.93	437	95.75	63.39	488	96.14	76.27
**30-30**	180	95.95	65.25	319	96.93	69.80	345	95.19	57.27	377	98.01	75.04
**45-5**	131	96.71	83.79	302	98.19	81.10	293	97.74	85.68	514	99.13	87.10
**45-11**	561	96.88	76.55	1107	97.15	80.04	974	97.73	84.1	1667	98.21	80.29
**45-16**	956	98.26	89.09	1089	97.57	85.57	688	97.46	80.65	1260	98.37	80.85
**45-23**	649	96.97	83.53	981	97.24	81.68	787	97.70	79.16	1593	98.52	81.54
**45-30**	662	97.42	80.71	972	95.84	80.72	950	96.60	79.51	1595	97.93	80.44

Table S4. Mean numbers (n = 3) of transcripts encoding key enzymes of plant polymer breakdown, which could be assigned on family level. Numbers are given in relation to five sampling time points for 30°C and 45°C.

	**Cellulase**	**Endo-1,4-beta-xylanase**	**Alpha-galactosidase**	**Beta-galactosidase**
**30-5**	98	139	139	76
**30-11**	148	157	146	125
**30-16**	102	115	113	81
**30-23**	127	143	119	95
**30-30**	90	86	76	73
**45-5**	71	108	78	73
**45-11**	192	316	268	200
**45-16**	321	303	133	167
**45-23**	291	320	160	222
**45-30**	273	272	189	238

Table S5. mRNA abundances related to acetoclastic and hydrogenotrophic methanogenesis.

Days	30°C			45°C		
	Avg. No.[1]	Acetoclastic methanogenesis (%)[2]	Hydrogenotrophic methanogenesis (%)[2]	Avg. No.[1]	Acetoclastic methanogenesis (%)[2]	Hydrogenotrophic methanogenesis (%)[2]
5	143±7	63.74±12.70	36.26±12.70	7±1	0.00	0.00
11	1461±55	61.32±1.21	38.68±1.21	64±2	1.23±2.14	98.77±2.14
16	773±28	23.67±0.25	76.33±0.25	377±92	0.24±0.42	99.76±0.42
23	993±100	36.84±3.16	63.16±3.16	4541±592	1.58±2.20	98.42±2.20
30	301±41	24.45±4.19	75.55±4.19	7030±246	0.04±0.06	99.96±0.06

[1]The average numbers of mRNA reads that could be assigned to GO biological processes.

[2]The calculation of mRNA transcript abundances related to acetoclastic and hydrogenotrophic methanogenesis was done as described previously [1]. Briefly, ID numbers of mRNA assigned to methanogenesis (based on SEED subsystems) were extracted from MEGAN6. The corresponding reads were extracted from mRNA fasta files by Python scripts. Respective subdatasets of methanogenesis-affiliated mRNA were queried against UNIREF90 [2] using the same search parameters as above. Resulting BLAST outputs were mapped against Gene Ontology database (GO, http://www.geneontology.org/). Mappings from UniRef90 to GO were done using available ID mappings (http://www.uniprot.org/downloads), which were stored as an indexed sqlite3 file to speed up database queries for mapping. Mapping was done by parsing the BLAST output with custom python scripts. GO terms of interest included "methanogenesis" (GO: 0015948), "methanogenesis from acetate" (GO:0019385), "methanogenesis from CO_2" (GO:0019386) and "methanogenesis from methanol" (GO:0019387).

Table S6. Sequencing statistics of 16S rRNA and 18S rRNA in metatranscriptomic datasets.

	Number of raw reads	Processed reads[1]	Mean length	16S rRNA	% processed reads	Phylum	% assigned 16S rRNA reads[2]	Family	% assigned 16S rRNA reads[3]	18S rRNA	% processed reads
**30-5-1**	5,157,486	2,856,173	269	1,271,937	44.38	1,115,370	87.69	1,078,411	84.78	39,986	1.40
**30-5-2**	4,898,199	2,546,808	270	1,153,298	45.13	990,525	85.89	960,511	83.28	33,618	1.32
**30-5-3**	5,567,775	3,053,755	268	1,283,983	41.90	1,123,762	87.52	1,092,079	85.05	36,034	1.18
**30-11-1**	5,841,253	2,830,631	264	1,161,423	41.03	1,043,591	89.85	1,009,340	86.91	48,970	1.73
**30-11-2**	3,837,685	2,227,500	275	974,645	43.76	874,327	89.71	844,602	86.66	34,749	1.56
**30-11-3**	4,935,749	2,725,427	275	1,192,535	43.76	1,070,545	89.77	1,033,060	86.63	38,974	1.43
**30-16-1**	4,471,932	2,460,365	265	1,032,737	41.97	933,510	90.39	902,724	87.41	42,072	1.71
**30-16-2**	4,680,086	2,584,457	269	1,081,607	41.85	976,598	90.29	944,966	87.37	46,262	1.79
**30-16-3**	4,295,407	2,462,277	273	1,039,666	42.22	936,865	90.11	906,317	87.17	46,045	1.87
**30-23-1**	4,217,125	2,425,631	277	977,834	40.31	874,862	89.47	845,160	86.43	60,156	2.48
**30-23-2**	3,678,228	2,154,501	281	879,928	40.84	786,955	89.43	759,416	86.30	50,631	2.35
**30-23-3**	5,804,136	3,221,256	268	1,312,790	40.75	1,169,184	89.06	1,126,848	85.84	70,223	2.18
**30-30-1**	4,174,218	2,327,420	275	1,096,239	47.10	978,445	89.25	940,964	85.84	64,470	2.77
**30-30-2**	3,908,117	2,167,254	269	1,014,124	46.79	904,203	89.16	871,763	85.96	63,067	2.91
**30-30-3**	5,068,017	2,836,755	276	1,359,766	47.93	1,216,284	89.45	1,171,280	86.14	79,429	2.80
**45-5-1**	3,704,178	2,238,540	279	986,359	43.92	832,403	84.39	815,064	82.63	43,204	1.93
**45-5-2**	4,311,769	2,620,347	284	1,160,016	44.12	980,055	84.49	966,487	83.32	47,952	1.83
**45-5-3**	3,810,324	2,203,865	274	948,596	42.89	811,919	85.59	792,515	83.55	41,212	1.87
**45-11-1**	3,450,734	2,202,296	284	898,866	40.69	783,834	87.20	722,747	80.41	39,421	1.79
**45-11-2**	3,698,776	2,247,423	281	1,007,074	44.67	877,938	87.18	834,671	82.88	33,487	1.49
**45-11-3**	4,236,734	2,563,393	282	1,053,962	40.99	915,902	86.90	858,476	81.45	46,397	1.81
**45-16-1**	4,940,751	3,193,179	282	1,375,518	42.96	1,234,278	89.73	1,157,170	84.13	59,393	1.86
**45-16-2**	4,182,105	2,348,243	274	1,236,052	52.41	1,081,836	87.52	1,051,450	85.07	28,179	1.20
**45-16-3**	4,022,571	2,518,427	281	1,115,953	44.17	1,001,756	89.77	937,576	84.02	47,850	1.90
**45-23-1**	3,854,992	2,475,424	284	1,152,296	46.38	1,015,314	88.11	985,121	85.49	35,399	1.43
**45-23-2**	4,832,386	3,071,109	282	1,518,034	49.30	1,344,176	88.55	1,302,786	85.82	39,617	1.29
**45-23-3**	4,514,183	2,864,927	280	1,372,265	47.75	1,243,508	90.62	1,220,824	88.96	40,968	1.43
**45-30-1**	3,765,434	2,420,768	282	1,141,068	46.96	984,071	86.24	965,689	84.63	24,692	1.02
**45-30-2**	5,130,372	3,241,492	279	1,448,868	44.55	1,314,697	90.74	1,279,181	88.29	47,002	1.45
**45-30-3**	4,058,488	2,576,315	284	1,211,190	46.85	1,037,697	85.68	1,009,741	83.37	31,946	1.24
**Total**	133,049,210	77,665,958		34,458,629		30,454,410		29,386,939		1,361,405	

[1] Average number (n=3) of quality-filtered and merged reads of 269-291 bp length.

[2,3] Average percentage (n=3) of bacterial and archaeal 16S rRNA reads that could be assigned on phylum and family levels.

Table S7. Domain-level distribution of SSU rRNA reads (means ± SE, n=3).

	**Archaea**	**Bacteria**	**Eukaryota**	**Others**
**30-5**	1.91±0.24	90.56±0.46	1.07±0.11	6.45±0.31
**30-11**	13.82±0.29	78.92±0.62	1.74±0.25	5.52±0.25
**30-16**	16.48±0.39	75.8±0.32	2.3±0.091	5.41±0.03
**30-23**	14.34±0.55	76.86±0.74	3.21±0.22	5.58±0.14
**30-30**	16.06±0.84	74.67±0.76	3.45±0.12	5.82±0.09
**45-5**	0.63±0.20	89.60±0.31	0.49±0.22	9.28±0.33
**45-11**	1.78±0.26	86.02±0.87	0.96±0.20	11.24±0.48
**45-16**	3.19±0.68	86.80±0.93	0.72±0.28	9.30±0.41
**45-23**	9.89±3.09	79.72±3.83	0.30±0.05	10.09±0.70
**45-30**	22.03±1.70	66.93±2.67	0.32±0.11	10.72±1.24

Table S8. Relative rRNA abundances of major eukaryal groups in total SSU rRNA reads.

	Archaeplastida	Opisthokonta /fungi	Excavata	SAR	Incertae Sedis	Amoebozoa
**30-5**	0.15±0.02	0.12±0.03	0.01	0.13±0.01	0.00	0.64±0.06
**30-11**	0.2±0.02	0.18±0.03	0.08±0.04	0.27±0.04	0.09±0.04	0.89±0.11
**30-16**	0.19	0.20±0.01	0.13±0.05	0.38±0.03	0.41±0.08	0.97±0.04
**30-23**	0.19±0.02	0.28±0.02	0.30±0.02	0.65±0.05	0.60±0.13	1.14±0.07
**30-30**	0.23±0.01	0.37±0.02	0.25±0.03	0.72±0.18	0.38±0.08	1.46±0.06
**45-5**	0.02±0.02	0.04±0.02	0.00	0.02±0.01	0.00	0.39±0.17
**45-11**	0.02±0.02	0.08±0.01	0.00	0.02±0.01	0.00	0.82±0.18
**45-16**	0.01±0.01	0.08±0.03	0.00	0.02±0.01	0.00	0.59±0.23
**45-23**	0.00	0.03±0.01	0.00	0.01	0.00	0.24±0.04
**45-30**	0.01±0.01	0.04±0.01	0.00	0.01	0.00	0.25±0.10

Table S9. Domain-level distribution of putative mRNA reads (means ± SE, n=3).

	Archaea	Bacteria	Eukaryota	Others
**30-5**	4.58±0.29	94.19±0.30	1.14±0.02	0.09±0.01
**30-11**	34.90±0.91	63.86±0.88	1.17±0.06	0.07±0.00
**30-16**	25.57±0.58	73.01±0.52	1.38±0.07	0.05±0.01
**30-23**	27.16±0.72	70.85±0.69	1.93±0.03	0.05±0.00
**30-30**	22.08±1.20	74.66±0.57	3.20±0.71	0.07±0.01
**45-5**	1.12±0.18	98.17±0.22	0.66±0.06	0.05±0.01
**45-11**	2.29±0.14	97.24±0.18	0.41±0.03	0.05±0.00
**45-16**	6.75±1.37	92.69±1.40	0.53±0.04	0.03±0.01
**45-23**	30.39±4.51	69.17±4.44	0.39±0.08	0.05±0.01
**45-30**	39.24±7.09	60.18±7.03	0.54±0.05	0.04±0.00

Table S10. Sequencing statistics of putative mRNA (only bacteria and archaea) in metatranscriptomic datasets.

	Reads assigned to mRNA	% processed reads[1]	Reads with NCBI nr hit[2]	% assigned[3]	Phylum	% assigned[4]	Family	% assigned[5]	SEED	% assigned[6]	KEGG	% assigned[7]	CAZy[8]	% assigned[8]
**30-5-1**	53,939	1.89	24,324	45.10	19,310	79.39	11,366	46.73	10,657	43.81	10,728	44.10	1,999	8.22
**30-5-2**	48,484	1.90	19,491	40.20	15,661	80.35	9,361	48.03	8,038	41.24	8,127	41.70	1,458	7.48
**30-5-3**	57,434	1.88	26,211	45.64	21,143	80.66	11,732	44.76	11,404	43.51	11,681	44.57	2,163	8.25
**30-11-1**	87,712	3.1	28,931	32.98	24,036	83.08	16,823	58.15	12,920	44.66	12,932	44.70	1,802	6.23
**30-11-2**	58,168	2.61	21,748	37.39	17,657	81.19	12,177	55.99	9,864	45.36	10,071	46.31	1,508	6.93
**30-11-3**	73,059	2.68	25,709	35.19	20,968	81.56	14,669	57.06	12,115	47.12	12,186	47.40	1,705	6.63
**30-16-1**	48,789	1.98	20,181	41.36	15,914	78.86	10,350	51.29	8,431	41.78	8,393	41.59	1,338	6.63
**30-16-2**	49,499	1.92	21,565	43.57	17,291	80.18	11,135	51.63	8,942	41.47	8,910	41.32	1,500	6.96
**30-16-3**	50,320	2.04	21,382	42.49	17,078	79.87	11,026	51.57	8,871	41.49	8,808	41.19	1,455	6.80
**30-23-1**	54,539	2.25	24,620	45.14	19,502	79.21	12,906	52.42	10,618	43.13	10,396	42.23	1,765	7.17
**30-23-2**	45,537	2.11	20,245	44.46	15,908	78.58	10,314	50.95	8,798	43.46	8,606	42.51	1,500	7.41
**30-23-3**	68,482	2.13	28,848	42.12	22,763	78.91	14,938	51.78	12,291	42.61	11,977	41.52	2,011	6.97
**30-30-1**	55,006	2.36	17,739	32.25	14,243	80.29	9,101	51.31	6,239	35.17	5,642	31.81	1,256	7.08
**30-30-2**	51,478	2.38	15,527	30.16	12,518	80.62	8,096	52.14	5,453	35.12	5,018	32.32	1,072	6.90
**30-30-3**	65,150	2.3	21,812	33.48	17,468	80.08	11,322	51.91	7,715	35.37	7,397	33.91	1,591	7.29
**45-5-1**	24,001	1.07	15,010	62.54	12,011	80.02	6,321	42.11	7,051	46.98	7,341	48.91	1,475	9.83
**45-5-2**	21,538	0.82	14,196	65.91	11,419	80.44	5,889	41.48	6,762	47.63	7,106	50.06	1,249	8.80
**45-5-3**	32,222	1.46	19,959	61.94	15,883	79.58	8,537	42.77	9,266	46.43	9,916	49.68	1,910	9.57
**45-11-1**	99,274	4.51	63,444	63.91	45,151	71.17	20,123	31.72	31,797	50.12	34,260	54.00	5,524	8.71
**45-11-2**	88,774	3.95	57,655	64.95	41,639	72.22	18,670	32.38	27,323	47.39	31,482	54.60	5,183	8.99
**45-11-3**	129,971	5.07	84,736	65.20	59,414	70.12	26,182	30.90	42,826	50.54	46,159	54.47	7,316	8.63
**45-16-1**	95,866	3.00	61,707	64.37	46,921	76.04	23,744	38.48	30,685	49.73	32,678	52.96	5,871	9.51
**45-16-2**	44,859	1.91	28,234	62.94	22,048	78.09	12,038	42.64	13,778	48.80	14,551	51.54	2,326	8.24
**45-16-3**	72,139	2.86	47,212	65.45	35,323	74.82	17,636	37.35	23,736	50.28	25,511	54.03	4,244	8.99
**45-23-1**	92,622	3.74	66,448	71.74	55,862	84.07	37,120	55.86	34,056	51.25	37,743	56.80	3,825	5.76
**45-23-2**	132,638	4.32	94,398	71.17	78,572	83.23	50,035	53.00	47,836	50.67	52,194	55.29	6,834	7.24
**45-23-3**	107,858	3.76	68,742	63.73	53,853	78.34	32,636	47.48	34,756	50.56	38,932	56.63	4,525	6.58
**45-30-1**	135,018	5.58	89,946	66.62	77,524	86.19	55,865	62.11	41,977	46.67	47,204	52.48	5,547	6.17
**45-30-2**	158,157	4.88	99,590	62.97	79,802	80.13	51,512	51.72	47,155	47.35	50,751	50.96	6947	6.98
**45-30-3**	142,716	5.54	93,321	65.39	79,046	84.70	53,955	57.82	45,127	48.36	50,298	53.90	5,609	6.01
**Total**	2,245,249		1,242,931		985,928		595,579		586,487		626,998		92,508	

[1] Percentage of putative mRNA reads in processed reads calculated by eliminating rRNA and non-coding small RNA reads via SORTMERNA 2.0.

[2] Total numbers of mRNA reads that had a homolog in NCBI nr database using BLASTx (e-value cutoff of 1e-5). mRNA reads with NCBI nr hits were subjected to taxonomic assignment and functional annotation.

[3] Total percentage of mRNA reads with NCBI nr hit in processed mRNA reads.

[4,5] Total percentage of bacterial and archaeal mRNA reads that could be assigned on phylum and family levels.

[6,7] Among mRNA reads with NCBI nr hit, the percentage of bacterial and archaeal mRNA reads that could be functionally annotated on SEED subsystems level 1 and KEGG categories using MEGAN6.

[8] Total percentage of genes that could be functionally annotated to dbCAN database

References

1. Wegner CE, Liesack W. Microbial community dynamics during the early stages of plant polymer breakdown in paddy soil. Environ Microbiol. 2016;18(9):2825-2842.

2. Suzek BE, Wang YQ, Huang HZ, McGarvey PB, Wu CH, Consortium U. UniRef clusters: a comprehensive and scalable alternative for improving sequence similarity searches. Bioinformatics. 2015;31(6):926-932.
