# Supplementary material for: Metatranscriptomics reveals a differential temperature effect on the structural and functional organization of the anaerobic food web in rice field soil
Source: Microbiome. 2018 Sep 19;6:169. doi: 10.1186/s40168-018-0546-9 (PMC6147125; doi:10.1186/s40168-018-0546-9)
Supplement: Supplementary file 3 — Detailed excursus on anaerobic polymer breakdown and the temperature-dependent expression of CAZymes. (PDF 87 kb) [file 40168_2018_546_MOESM3_ESM.pdf]

# Metatranscriptomics reveals a differential temperature effect on the structural and functional organization of the anaerobic food web in rice field soil

Jingjing Peng[1], Carl-Eric Wegner[2], Qicheng Bei[1], Pengfei Liu[1], Werner Liesack[1]

**Supporting information:**

**Anaerobic breakdown of complex plant polymers**

***Anaerobic breakdown of complex plant polymers***

Although the CAZy database (http://www.cazy.org; [1]) is an invaluable resource, it suffers from two major shortcomings in that the underlying sequence information and respective CAZyme family definitions are not easily accessible. The usage of dbCAN as complementary resource allowed us to bypass these problems and to make use of available CAZy to enzyme commission (EC) number mappings to define functional modules that comprise enzyme functions of interest. Here the method-inherent limitation is that the same EC number can be linked to multiple but similar functions. We accepted this shortcoming, given that CAZyme annotations are generally prone to some noise due to both modularity of CAZymes and in part broad substrate ranges. CAZyme modularity often relates to glycosyl hydrolase (GH) modules being combined with carbohydrate-binding modules (CBM), which are mostly coexpressed. Available CAZyme to EC mappings compensated for this modularity and allowed us to confidently annotate CBM-affiliated mRNA reads to enzyme functions of associated GH domains.

To identify microbial groups and key enzymes involved in polymer breakdown in rice field soil, we surveyed our mRNA datasets against dbCAN. The heterogenous composition of rice straw as a complex biopolymer (cellulose 32-37%, hemicellulose 29-37%, and lignin 5-15% [2, 3]) was well reflected by the CAZyme expression profiles. Totally, 92,508 mRNA reads were identified to encode CAZymes. Of these, 75,438 reads were assigned to enzyme functions related to the degradation of cellulose, xylan, other hemicelluloses, and chitin. These were primarily GHs and CBMs (GH/CBM) (Figure 3).

Both cellulose and xylan are not only the most abundant polysaccharides on earth, but also the main components of rice straw [4, 5] with xylan being the main constituent of hemicellulose. Correspondingly, transcripts involved in degrading cellulose, xylan, and other hemicelluloses

collectively contributed most to our CAZyme-affiliated mRNA reads. Pectin is only a minor component (2-3%) of rice straw [6] and mRNA reads related to its degradation accounted for < 1% of total GH/CBM transcripts. By contrast, GH/CBM transcripts related to chitin degradation made - for a particular polymer – the greatest contribution to our CAZyme-affiliated mRNA reads (Additional file 1: Tables S3 and S4). This was unexpected given that chitin was present only in minor amounts. The overrepresentation of chitinase transcripts among the CAZyme-affiliated mRNA may have several reasons as discussed below.

On phylum level, the assignment rate of GH/CBM transcripts for each of the sampling time points (both 30°C and 45°C) was always greater than 94% (Additional file 1: Table S3). The vast majority of GH/CBM transcripts was affiliated to *Firmicutes*, in particular at 45°C (Fig. 3a). Both temperature and incubation time had a significant impact on the taxonomic composition of the GH/CBM transcript pool. At 30°C, the *Firmicutes* abundance steadily decreased till day 30, while that of the *Proteobacteria, Planctomycetes, Bacteroidetes,* and *Ignavibacteriae* collectively increased. Members of these phyla are involved in polymer breakdown [7, 8]. In particular, *Bacteroidetes* are well known for their important role in polymer degradation [9]. Relative to their contribution to total bacterial mRNA, non-*Firmicutes* populations were overpresented in the GH/CBM transcript pool (21.5% to 37.2% [total bacterial mRNA] compared to 18.5% to 74.6% [bacterial GH/CBM transcripts]). This is most evident for the phylum *Bacteroidetes* that after *Firmicutes*, contributed second most to the bacterial GH/CBM transcript pool (Fig. 3a). Conclusively, the degradation of rice straw at 30°C involves a complex functional interaction of diverse bacterial taxa, in which the contribution of non-*Firmicutes* populations strongly increases with incubation time. By contrast, at 45°C, *Firmicutes* was the dominant taxon throughout incubation time, in good correspondence to its contribution to total bacterial mRNA (Fig. 3a and Additional file 1: Figures S4, S5).

On family level, the assignment rate of GH/CBM transcripts depended on polymer, sampling time point and temperature, and varied between 57% and 89% (Additional file 1: Table S3). The taxonomic composition of the GH/CBM transcripts greatly differed for each of the polymers between 30°C and 45°C (Additional file 1: Fig. S5). Unexpectedly, at 30°C, methanogens of the families *Methanosarcinaecae* and, to lower extent, *Methanoregulaceae* contributed to the GH/CBM transcripts pools of all the polymers, but most pronounced to cellulose degradation. No or only minor amounts of methanosarcinal GH/CBM transcripts were detected at 45°C. GH/CBM transcripts affiliated with methanogens at 45°C were primarily related to the *Methanocellaceae*. Members of this family made a major contribution to the GH/CBM transcripts involved in degrading cellulose and "other hemicelluloses" on day 30 (Additional file 1, Fig. S5a,b). Our study is the first report of a potential contribution of methanogens to polymer hydrolysis, while the expression of glycosyl transferases by methanogens is well confirmed [10]. In fact, there exists no published evidence for the potential of methanogens to express GHs involved in the degradation of cellulase, xylan, or other hemicelluloses. Therefore, we collected all the available complete methanogen genome sequences from NCBI RefSeq and queried them against dbCAN using DIAMOND, applying an e-value threshold of 1e-3. The 88 methanogen genomes comprised 214,249 coding sequences, of which 13,975 had a hit in dbCAN (6.5%). A total of 2691 sequences (1.3%) showed hits for GHs. The dbCAN consortium recently made a webserver available that facilitates CAZyme sequence annotation. An e-value threshold of 1e-102 is suggested for DIAMOND-based querying. Applying this very stringent threshold and making a 100% sequence identity mandatory reduces the number of hits against GHs to 0.14% of total methanogen genes. These high-quality hits included GH families 113, 13, 130, 133, 15, 16, 18, 23, 26, 38, 39, 46, 57, and 77. Some of these GH families are linked to complex polysaccharide cleavage; for instance GH18, 23 and 46 with chitin turnover, and GH26 and 39 with xylan

breakdown. Hits for cellulases and related endo-glucanases mostly showed sequence identities below 100% (see Additional File 2). Taken together, the functional annotation of methanosarcinal mRNA transcripts as encoding extracellular GHs involved in polymer hydrolysis has to be interpreted with caution, but calls for further research.

Among *Firmicutes*, various family-level groups contributed to the decomposition of rice straw at both 30°C and 45°C: *Clostridiaceae*, *Ruminococcaceae*, *Lachnospiraceae*, *Bacillaceae*, *Paenibacillaceae, Symbiobacteriaceae*, and *Peptococcaceae* (Additional file 1: Fig. S5). Previous studies have shown that members of these families are involved in degrading cellulose, xylan, other hemicelluloses, and chitin (e.g., [11, 12]). Key enzymes involved rice straw degradation are cellulase, endo-1,4-beta-xylanase, and alpha- and beta-galactosidases. Four family-level groups were detected to contribute to the production of all the key enzymes, with transcripts affiliated to *Methanosarcinaceae* being predominant at 30°C, and *Clostridiaceae*, *Ruminococcaceae*, and *Paenibacillaceae* being most abundant in the GH/CBM transcript pool at 45°C. Transcripts affiliated with *Methanosarcinaceae* were overrepresented in the family-level patterns at 30°C. This is due to the fact that most of the GH/CBM transcripts affiliated with the *Proteobacteria*, *Planctomycetes*, *Bacteroidetes*, and *Ignavibacteriae* could not be assigned on family level or the families were below the abundance threshold (<0.5%).

Cellulase is the key enzyme in cellulose degradation. It catalyzes the first step in cellulose hydrolysis that involves the splitting of cross-links between glucan chains by endoglucanases. Cellulase transcripts were highly abundant, followed by those encoding cellulose 1,4-beta-cellobiosidase. Both transcript species showed the same abundance dynamics over time. These differed between 30°C and 45°C. Their relative abundance did not majorly change between days 5 and 30 at 30°C, but showed a peak abundance on day 16 at 45°C (Fig. 3b). Cellobiose is the

major product of cellulose degradation. Notably, the transcripts encoding cellobiose phosphorylase were more abundant at 45°C than at 30°C throughout incubation time (Fig. 3b). This enzyme catalyzes the reversible phosphate-dependent hydrolysis of cellobiose. It was shown to exhibit a higher activity at 50°C than below 40°C [13] and to be widely distributed among the *Clostridia*, such as *Clostridium stercorarium* [14] and *Ruminococcus flavefaciens* [13].

Xylan is known to have a strong affinity to cellulose [15]. Its strong adsorption to cellulose is believed to act as the limiting factor in the enzymatic hydrolysis of cellulose [16]. The abundance dynamics of cellulase and endo-1,4-beta-xylanase transcripts were nearly identical, suggesting that the degradation of cellulose and xylan was highly interrelated (Fig. 3b). This is most obvious for 45°C. A coordinated degradation activity is further evidenced by the highly similar taxonomic patterns observed for cellulase and endo-1,4-beta-xylanase transcripts over time, with a peak abundance of the *Clostridicaceae* and *Ruminoccocaceae* collectively on day 16. In addition to endo-1,4-beta-xylanase, the abundance dynamics of transcripts encoding acetylxylan esterase differed between 30°C and 45°C, with much higher relative transcript levels at 30°C. Thus, temperature had a determinative effect on the transcript levels of particular GHs. It has been shown that the addition of xylanases and acetylxylan esterases significantly improves the performance of cellulase, with an increase in cellulose conversion in many lignocellulosic materials [17, 18]. In our study, the interrelation between cellulolytic and xylanolytic transcript dynamics was evident for both temperatures, but in particular for 45°C.

In addition to xylan, hemicelluloses include xyloglucans, mannans and glucomannans, and β-(1→3, 1→4) – glucans [5]. Alpha- and beta-galactosidases are the two most abundant hemicellulose-degrading enzymes. Their transcript dynamics clearly differed between 30°C and 45°C. At 30°C, the alpha-galactosidase transcripts showed a significantly greater abundance

throughout incubation than those encoding beta-galactosidase. However, at 45°C, the transcript abundances showed the opposite trend during the later incubation stages. These temperature-dependent differences in relative transcript levels presumably relate to the microorganisms involved in hemicellulose degradation. In fact, four family-level groups that greatly contributed to the production of alpha- and beta-galactosidases – *Symbiobacteriaceae*, *Methanosarcinaceae*, *Peptococcaceae,* and *Ruminoccocaceae* - varied in their abundance as a function of enzyme type, temperature, and incubation time (Additional file 1: Fig. S5c). Intriguingly, the *Symbiobacteriaceae* made a major contribution to the pool of GH transcripts encoding alpha-galactosidase, but not to any of the other key enzymes (Additional file 1: Fig. S5c). The ability to produce alpha-galactosidase appears to be an adaptive trait of particular *Symbiobacterium* spp. Isolated from compost, the thermophilic nonspore-forming species *Symbiobacterium toebii* was shown to produce alpha-galactosidase, but not beta-galactosidase [19]. Other *Symbiobacterium* spp., however, are negative for both alpha-galactosidase and beta-galactosidase, such as *S. ostreiconchae*, *S. turbinis*, and *S. terraclitae* [20].

Chitinase transcripts were the most abundant individual type of GHs, with relatively stable mRNA abundances throughout incubation at both 30°C and 45°C. Other genes known to be involved in chitinase degradation, such as N-acetylglucosaminidase, chitin-deacetylase, N-acetylglucosaminylphosphatidylinositol deacetylase, were expressed only on a very low level (Fig. 3b). A large proportion of chitinase transcripts was affiliated with the *Clostridiaceae* (Additional file 1: Fig. S5a,b). A recent whole-genome transcriptome study on a plant litter-degrading *Clostridium* species has shown that chitinase transcripts are highly expressed during cellulolytic breakdown [21]. One may speculate that CAZymes annotated as chitinases are involved in degrading cellulose due to cross-substrate specificity. The cellular coexpression of cellulases and chitinases may be a phenomenon that is widely distributed not only among

*Clostridia*, but also among other microbial groups. In our study, it would explain why chitinase transcripts exhibit a stable and consistently high abundance during plant polymer breakdown.

## References


1. Lombard V, Ramulu HG, Drula E, Coutinho PM, Henrissat B. The carbohydrate-active enzymes database (CAZy) in 2013. Nucleic Acids Res. 2014;42(D1):490-495.
2. Tsutsuki K, Ponnamperuma FN. Behavior of anaerobic decomposition products in submerged soils - effects of organic material amendment, soil properties, and temperature. Soil Sci Plant Nutr. 1987;33(1):13-33.
3. Watanabe A, Katoh K, Kimura M. Effect of rice straw application on $CH_4$ emission from paddy fields. II. Contribution of organic constituents in rice straw. Soil Sci Plant Nutr. 1993;39(4):707-712.
4. Podkaminer KK, Guss AM, Trajano HL, Hogsett DA, Lynd LR. Characterization of xylan utilization and discovery of a new endoxylanase in *Thermoanaerobacterium saccharolyticum* through targeted gene deletions. Appl Environ Microbiol. 2012;78(23):8441-8447.
5. Scheller HV, Ulvskov P. Hemicelluloses. Annu Rev Plant Biol. 2010;61:263-289.
6. Zhao JS, Fu CG, Yang ZY. Integrated process for isolation and complete utilization of rice straw components through sequential treatment. Chem Eng Commun. 2008;195(9):1176-1183.
7. Ivanova AA, Wegner CE, Kim Y, Liesack W, Dedysh SN. Identification of microbial populations driving biopolymer degradation in acidic peatlands by metatranscriptomic analysis. Mol Ecol. 2016;25(19):4818-4835.
8. Kadnikov VV, Mardanov AV, Podosokorskaya OA, Gavrilov SN, Kublanov IV, Beletsky AV, Bonch-Osmolovskaya EA, Ravin NV. Genomic analysis of *Melioribacter roseus*, facultatively anaerobic organotrophic bacterium representing a novel deep lineage within *Bacteriodetes*/*Chlorobi* group. PloS ONE. 2013;8(1).
9. Thomas F, Hehemann JH, Rebuffet E, Czjzek M, Michel G. Environmental and gut *Bacteroidetes*: the food connection. Front Microbiol. 2011;2.

10. Magidovich H, Eichler J. Glycosyltransferases and oligosaccharyltransferases in Archaea: putative components of the N-glycosylation pathway in the third domain of life. FEMS Microbiol Lett. 2009;300(1):122-130.

11. Bandounas L, Wierckx NJP, de Winde JH, Ruijssenaars HJ. Isolation and characterization of novel bacterial strains exhibiting ligninolytic potential. BMC Biotechnol. 2011;11.

12. Wang YX, Liu Q, Yan L, Gao YM, Wang YJ, Wang WD. A novel lignin degradation bacterial consortium for efficient pulping. Bioresource Technol. 2013;139:113-119.

13. Hamura K, Saburi W, Abe S, Morimoto N, Taguchi H, Mori H, Matsui H. Enzymatic characteristics of cellobiose phosphorylase from *Ruminococcus albus* NE1 and kinetic mechanism of unusual substrate inhibition in reverse phosphorolysis. Biosci Biotech Bioch. 2012;76(4):812-818.

14. Reichenbecher M, Lottspeich F, Bronnenmeier K. Purification and properties of a cellobiose phosphorylase (CepA) and a cellodextrin phosphorylase (CepB) from the cellulolytic thermophile *Clostridium stercorarium*. Eur J Biochem. 1997;247(1):262-267.

15. Simmons TJ, Mortimer JC, Bernardinelli OD, Poppler AC, Brown SP, Deazevedo ER, Dupree R, Dupree P. Folding of xylan onto cellulose fibrils in plant cell walls revealed by solid-state NMR. Nat Commun. 2016;7.

16. Penttila PA, Varnai A, Pere J, Tammelin T, Salmen L, Siika-aho M, Viikari L, Serimaa R. Xylan as limiting factor in enzymatic hydrolysis of nanocellulose. Bioresource Technol. 2013;129:135-141.

17. Garcia-Aparicio MP, Ballesteros M, Manzanares P, Ballesteros I, Gonzalez A, Negro MJ. Xylanase contribution to the efficiency of cellulose enzymatic hydrolysis of barley straw. Appl Biochem Biotech. 2007;137:353-365.

18. Zhang JH, Siika-aho M, Tenkanen M, Viikari L. The role of acetyl xylan esterase in the solubilization of xylan and enzymatic hydrolysis of wheat straw and giant reed. Biotechnol Biofuels. 2011;4.

19. Sung MH, Bae JW, Kim JJ, Kim K, Song JJ, Rhee SK, Jeon CO, Choi YH, Hong SP, Lee SG, Ha JS, Kang GT. *Symbiobacterium toebii* sp nov., commensal thermophile isolated from Korean compost. J Microbiol Biotechn. 2003;13(6):1013-1017.

20. Shiratori-Takano H, Akita K, Yamada K, Itoh T, Sugihara T, Beppu T, Ueda K. Description of *Symbiobacterium ostreiconchae* sp nov., *Symbiobacterium turbinis* sp nov and *Symbiobacterium*

*terraclitae* sp nov., isolated from shellfish, emended description of the genus *Symbiobacterium* and proposal of *Symbiobacteriaceae* fam. nov. Int J Syst Evol Micr. 2014;64:3375-3383.

21. Tolonen AC, Cerisy T, El-Sayyed H, Boutard M, Salanoubat M, Church GM. Fungal lysis by a soil bacterium fermenting cellulose. Environ Microbiol. 2015;17(8):2618-2627.
